# Association of Hospitalization and Mortality Among Patients Initiating Dialysis With Hemodialysis Facility Ownership and Acquisitions

**DOI:** 10.1001/jamanetworkopen.2019.3987

**Published:** 2019-05-17

**Authors:** Kevin F. Erickson, Bo Zhao, Jingbo Niu, Wolfgang C. Winkelmayer, Jay Bhattacharya, Glenn M. Chertow, Vivian Ho

**Affiliations:** 1 Section of Nephrology, Baylor College of Medicine, Houston, Texas; 2 Center for Innovations in Quality, Effectiveness, and Safety, Baylor College of Medicine, Houston, Texas; 3 Baker Institute for Public Policy, Rice University, Houston, Texas; 4 Center for Primary Care and Outcomes Research, Stanford University School of Medicine, Stanford, California; 5 Division of Nephrology, Stanford University School of Medicine, Palo Alto, California

## Abstract

**Importance:**

Mergers and acquisitions among health care institutions are increasingly common, and dialysis markets have undergone several decades of mergers and acquisitions.

**Objective:**

To examine the outcomes of hemodialysis facility acquisitions independent of associated changes in market competition resulting from acquisitions.

**Design, Setting, and Participants:**

Cohort study using difference-in-differences (DID) analyses to compare changes in health outcomes over time among in-center US dialysis facilities that were acquired by a hemodialysis chain with facilities located nearby but not acquired. Multivariable Cox proportional hazards regression models and negative binomial models with predicted marginal effects were developed to examine health outcomes, controlling for patient, facility, and geographic characteristics. All facility ownership types were examined together and stratified analyses were conducted of facilities that were independently owned and chain owned prior to acquisitions. The study was conducted from January 2001 to September 2015; 174 905 patients starting in-center dialysis in the 3 years before and following dialysis facility acquisitions were included. Data were analyzed from March 2017 to December 2018.

**Exposures:**

Acquisition by a hemodialysis chain.

**Main Outcomes and Measures:**

Twelve-month hazard of death and hospital days per patient-year were the primary outcomes.

**Results:**

Of the 174 905 patients included in the study, 79 705 were women (45.6%), 24 409 (14.0%) were of Hispanic ethnicity, 61 815 (35.3%) were black, 105 272 (60.2%) were white, and 1247 (0.7%) were Native American. Mean (SD) age was 65 (15) years. Before acquisitions, adjusted mortality and hospitalization rates were 10% (95% CI, −16% to −5%) and 2.9 days per patient-year (95% CI, −3.8 to −2.0) lower, respectively, at independently owned facilities that were acquired compared with those that were not acquired, while hospitalization rates were 0.7 days (95% CI, −1.2 to −2.0) lower at chain-owned facilities that were acquired compared with those that were not acquired. In stratified analyses of independently owned facilities, mortality decreases were smaller at acquired (−8.4%; 95% CI, −14% to −25%) vs nonacquired (−20.3%; 95% CI, −25.8% to −14.3%) facilities (DID *P* < .001). Similarly, hospitalization rates did not change at acquired facilities and decreased by 2.6 days per patient-year (95% CI, −3.6 to −1.7 days) at nonacquired facilities (DID *P* < .001). Acquisitions were not associated with changes in health outcomes at chain-owned facilities. Slower reductions in mortality and hospitalization rates at independently owned facilities contributed to significant differences in hospitalizations (−2.0 days; 95% CI, −2.5 to −1.6, at nonacquired vs 0.9 days; 95% CI, −1.3 to −0.5, at acquired facilities; DID, *P* < .001) across all ownership types but not mortality (DID, *P* = .28) with regard to acquisitions.

**Conclusions and Relevance:**

Acquisition of independently owned dialysis facilities by larger dialysis organizations was associated with slower decreases in mortality and hospitalization rates, as nonacquired facilities appeared to experience more rapid improvements in outcomes over time.

## Key Points

**Question:**

Are acquisitions by dialysis facility chains associated with patient health outcomes?

**Findings:**

In a cohort study using difference-in-differences analyses of US patients initiating hemodialysis from 2001 to 2015, hospitalization days per patient-year and mortality decreased over time in most comparison groups. However, decreases in hospitalizations were significantly slower at acquired vs nonacquired facilities; observed acquisition results were largely associated with changes at independently owned facilities that were acquired by dialysis chains, where acquired facilities had significantly slower decreases in both mortality and hospitalization rates than nonacquired facilities.

**Meaning:**

Acquisition of independently owned dialysis facilities by dialysis chains suggests slower decreases in mortality and hospitalization rates than would have otherwise occurred.

## Teaser

This study uses difference-in-differences analysis to evaluate the association between acquisitions of independent hemodialysis facilities by a hemodialysis chain and hospitalization and mortality rates among adult patients receiving dialysis.

## Introduction

In 1991, a report by the Institute of Medicine on the quality of US dialysis care called for monitoring the consequences of a changing mix of dialysis facilities.^1^ In the years since that report, ongoing mergers and acquisitions—wherein one dialysis facility chain purchases independent dialysis facilities or other, smaller dialysis chains—have moved the dialysis industry toward ownership by a small number of large organizations.^2^ Between 2004 and 2006, the acquisition of 2 dialysis chains by 2 larger, for-profit national dialysis chains involved nearly 1000 dialysis facilities treating approximately 70 000 patients,^3,4^ or 10% of all US patients receiving dialysis.^5^ Mergers and acquisitions have continued since then.^6,7^ Despite the broad scope of change in the composition of owners in dialysis facility markets, the effects of hemodialysis facility acquisitions on the cost and quality of dialysis care are unknown and may vary by organizational and ownership status.^8,9,10,11,12,13^

Mergers and acquisitions among health care institutions can have varying effects on health care costs and patient health outcomes at acquired organizations. When larger organizations more effectively provide specialized, integrated care and invest in quality improvement, the acquisition of smaller organizations may improve both the efficiency and quality of care delivered.^14,15,16,17,18^ In contrast, acquisitions by lower quality organizations could lead to worse health outcomes at the acquired organizations. Apart from the direct consequences of acquisitions, reduced market competition resulting from consolidation may lead to higher prices and, in some instances, lower quality of care.^19^ Whether mergers and acquisitions yield net improvements in patient health and health care costs depends on the relative magnitudes of these counterbalancing factors.^20,21^

In this study, we examined whether acquisitions by dialysis chains are associated with mortality and hospitalization rates among patients initiating in-center hemodialysis. We focused our analysis on the estimated results of mergers and acquisitions while accounting for associated changes in local market competition. We assessed acquisitions affecting all dialysis facilities and conducted stratified analyses of facilities that were independently owned vs being part of a chain prior to acquisition.

## Methods

### Data Sources

We identified patients initiating in-center hemodialysis (incident patients) between January 2001 and September 2015 from a national end-stage renal disease (ESRD) registry.^22^ Patient comorbidity information and ethnicity came from the Centers for Medicare & Medicaid Services (CMS) Medical Evidence Report (CMS-2728), which nephrologists and their staff complete for all patients at the onset of ESRD. We obtained information about patient race from Medicare enrollment. Information about large dialysis facility chain ownership came from annual facility surveys, and ownership by smaller chains and independent facilities came from CMS Dialysis Facility Compare. We linked patient zip codes to census-based rural and urban commuting area codes^23^ and data from the Dartmouth Atlas Project^24^ to identify population density and assign patients to hospital service areas (HSAs). We analyzed data from March 2017 to December 2018.

This study followed the Strengthening the Reporting of Observational Studies in Epidemiology (STROBE) reporting guideline for cohort studies. This project was approved by an institutional review board at Baylor College of Medicine, which waived informed consent owing to minimal risk to patients from the use of previously collected, deidentified data.

### Study Outcomes

The main study outcomes were hazard of death and hospital days per patient-year. We followed up patients for 12 months beginning on their fourth month of dialysis because this is a time of high acuity when changes in care delivery associated with acquisitions would most likely affect patient health outcomes. Most US patients with ESRD qualify for Medicare on their fourth dialysis month. Because we could only ascertain hospitalizations from Medicare claims, we restricted our analysis to patients with Medicare as their primary payer on the first day of their fourth dialysis month.

### Study Design and Exposure

We used difference-in-differences (DID) models to estimate the health outcomes of acquisitions by dialysis facility chains. In the context of the DID analysis, we compared changes over time in mortality rates and days hospitalized at facilities that underwent acquisition with those at nearby facilities that were not acquired. The DID model reduces bias by using each facility as its own control, which accounts for unobserved characteristics at acquired facilities that do not change over time and contribute to health outcomes. The difference in the change in outcomes among these comparison groups represents an estimate of the effect of acquisitions. We used Cox proportional hazards regression models when examining time to death and negative binomial models with predicted marginal effects when examining hospital days per patient-year.

### Identifying Acquired Facilities and Nearby Controls

We tracked dialysis facility ownership each year. We assigned facilities that reported changing ownership in a given year to the acquisition comparison group if ownership was otherwise unchanged in the 3 years before and after the reported ownership change. We used dialysis facility addresses to identify the HSA where each facility in the acquisition comparison group was located immediately before ownership change and identified all facilities located in the same HSAs that did not report a change in ownership in the 3 years before and after the acquisition. These facilities served as nearby controls in the DID analysis. Although the control facilities were not directly involved in acquisitions, their geographic proximity makes them similar in many ways to acquired facilities, including experiencing similar changes in local market concentration resulting from acquisitions.^2,25,26^ Our process of selecting acquired facilities and nearby controls for comparison yielded 10 acquisition cohorts distinguished by the calendar year of dialysis chain acquisition. The same facility could appear in multiple cohorts.

### Patient Selection and Comparison Groups

For each acquisition cohort, we identified all patients initiating hemodialysis at selected facilities in the 3 years before and after acquisition. Because patients frequently change dialysis facilities early in the course of their therapy (eMethods in the Supplement), we assigned patients to facilities based on where they received dialysis on their 90th day of ESRD. Although patients receiving peritoneal dialysis or home hemodialysis could also be affected by facility acquisition, the bulk of their treatment is performed at home. Our study focused on patients receiving in-center hemodialysis whose frequent contact with dialysis facilities makes them particularly susceptible to changes in health care delivery resulting from ownership changes.

For patients who initiated dialysis at an acquired facility, we compared outcomes for patients treated before the facility was acquired with those treated after the acquisition. In addition to examining the estimated outcome of acquisitions on all facility types, we compared outcomes separately among facilities that were independently owned vs chain owned before acquisitions.

### Patient Covariates and Additional Population Restrictions

**Figure 1.**
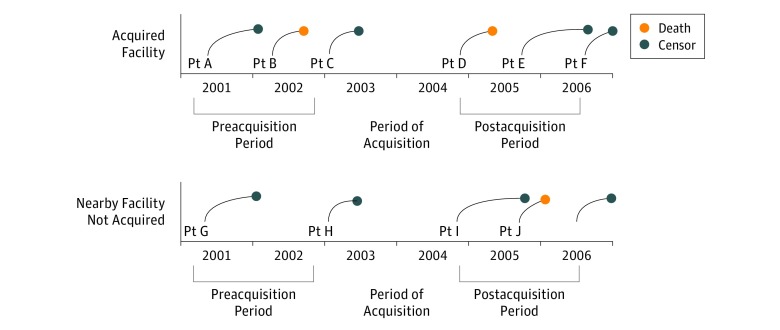
Method of Cohort Selection for 1 Hypothetical Cohort This schematic illustrates cohort selection in 1 acquisition year (acquisitions occurring between 2003 and 2004). An identical approach was used in subsequent acquisition years: 2004, 2005, 2006, 2007, 2008, 2009, 2010, 2011, 2012, and 2013. Before analysis, we combined the 10 acquisition cohorts spanning the study period and included dummy indicators representing each cohort in regression models. In rare instances when patients appeared more than once in the combined cohort, we used a predefined algorithm to assign them to only 1 cohort. When examining hospital days, we divided each patient follow-up period into up to 12 intervals spanning 30 days each and ascertained days spent in the hospital during each 30-day interval. We excluded 30-day intervals when patients were censored along with all subsequent 30-day intervals, with the following exception: when patients died, we included the 30-day interval of death since patients are frequently hospitalized before death. Pt indicates patient.

In all analyses we controlled for patient, geographic, and hemodialysis facility characteristics listed in the Table. These factors included age, sex, and race/ethnicity, which are associated with health outcomes in patients receiving hemodialysis. We used multiple imputation to account for missing data on serum albumin level (26.7%), hemoglobin level (8.4%), and Quételet (body mass) index (0.9%).^27,28^ When examining mortality, we censored patients if they moved to a new HSA for more than 60 days, recovered kidney function, or received a kidney transplant. When examining hospitalizations, we censored patients if they moved, recovered kidney function, lost Medicare coverage, or died. Our data only enabled us to identify the year that an acquisition occurred. To accurately assign patients to preacquisition and postacquisition groups, we excluded patients starting hemodialysis in the 6 months before and after January 1 of the year when ownership changes occurred and censored patients receiving hemodialysis during this transition period. As a result, the preacquisition and postacquisition periods included different patients (Figure 1).

**Table.** Patient Characteristics Involving All Facility Types^a^

Characteristic	Before Acquisition		After Acquisition		*P* Value^b^
	Acquired (n = 45 636)	Not Acquired (n = 49 107)	Acquired (n = 39 355)	Not Acquired (n = 40 807)	
Age, No. (%), y					
18-49	6906 (15.1)	8902 (18.1)	6045 (15.4)	7469 (18.3)	.82
50-64	11268 (24.7)	13646 (27.8)	10531 (26.8)	11913 (29.2)	.07
65-74	12797 (28.0)	13033 (26.5)	10638 (27.0)	10405 (25.5)	.87
≥75	14665 (32.1)	13526 (27.5)	12141 (30.8)	11020 (27.0)	.13
Women, No. (%)	21046 (46.1)	22730 (46.3)	17810 (45.3)	18119 (44.4)	.03
Race/ethnicity, No. (%)^c^					
White^d^	29831 (65.4)	26971 (54.9)	25811 (65.6)	22659 (55.5)	.45
Black^d^	13717 (30.1)	20006 (40.7)	11887 (30.2)	16205 (39.7)	.01
Native American	523 (1.1)	210 (0.4)	378 (1.0)	136 (0.3)	.58
Other	1565 (3.4)	1920 (3.9)	1279 (3.2)	1807 (4.4)	<.001
Hispanic^d^	4986 (10.9)	8266 (16.8)	4416 (11.2)	6741 (16.5)	.06
Medicaid eligible, No. (%)	17191 (37.7)	20415 (41.6)	15211 (38.7)	17544 (43.0)	.40
Uninsured, No. (%)^d^	3839 (8.4)	5798 (11.8)	3483 (8.9)	4725 (11.6)	.02
Median household income, $10 000, mean (SD)^e^	4.5 (2.2)	4.3 (2.4)	4.5 (2.2)	4.4 (2.4)	.06
Poverty rate per 100 residents, mean (SD)^d^^,^^f^	16.7 (14.3)	19.3 (17.3)	16.6 (14.1)	18.6 (17.0)	.02
Health status					
Cancer, No. (%)	3097 (6.8)	3021 (6.2)	3009 (7.6)	2659 (6.5)	.08
Heart failure, No. (%)	16382 (35.9)	16124 (32.8)	13902 (35.3)	12960 (31.8)	.23
Cerebrovascular disease, No. (%)	4820 (10.6)	4766 (9.7)	4079 (10.4)	3972 (9.7)	.45
Diabetes, No. (%)	25033 (54.9)	26935 (54.8)	21859 (55.5)	23047 (56.5)	.05
Coronary disease, No. (%)^d^	12977 (28.4)	11020 (22.4)	8729 (22.2)	7313 (17.9)	.03
Drug or alcohol abuse, No. (%)	901 (2.0)	1228 (2.5)	918 (2.3)	1094 (2.7)	.12
Immobility, No. (%)	2439 (5.3)	2964 (6.0)	2742 (7.0)	3171 (7.8)	.78
eGFR, median (IQR), mL/min/1.73 m^2^^g^	8.2 (5.4)	7.8 (5.3)	8.7 (5.6)	8.3 (5.5)	.29
Serum albumin level, median (IQR), g/dL^d^^,^^h^	3.2 (0.9)	3.1 (0.9)	3.2 (0.9)	3.2 (0.9)	.35
Hemoglobin level, median (IQR), g/dL^i^	10.0 (2.2)	9.8 (2.2)	9.9 (2.1)	9.5 (2.1)	<.001
BMI, median (IQR)^j^	26.6 (8.6)	26.7 (9.1)	27.4 (9.4)	27.3 (9.4)	<.001
Population density, %					
Metropolitan^d^	36542 (80.1)	46999 (95.7)	31316 (79.6)	39075 (95.8)	.25
Micropolitan^d^	5002 (11.0)	1056 (2.2)	4534 (11.5)	945 (2.3)	.70
Rural and small town^d^	4092 (9.0)	1052 (2.1)	3505 (8.9)	787 (1.9)	.06
HHI, median (IQR)^d^	0.4 (0.3)	0.3 (0.1)	0.5 (0.3)	0.4 (0.1)	<.001
Distance to facility, median (IQR), km	7.0 (13.3)	6.3 (8.6)	6.8 (13.7)	6.2 (8.5)	.03

Abbreviations: BMI, body mass index (calculated as weight in kilograms divided by height in meters squared); eGFR, estimated glomerular filtration rate; HHI, Hefindahl-Hirschman Index; IQR, interquartile range.

SI conversion factors: To convert albumin to grams per liter, multiply by 10; hemoglobin to grams per liter, multiply by 10.

^a^ eTable 3 and eTable 4 in the Supplement include baseline characteristics of patients at independently owned and chain-owned facilities, respectively.

^b^ Represents the statistical significance of interaction terms where characteristics of interest are a function of case vs control, before vs after acquisition, and the interaction between case and after acquisition.

^c^ Groupings obtained from the US Renal Data System.

^d^ Greater than 10% standardized difference in characteristics in the preacquisition period.

^e^ Range, $3000-$234 000.

^f^ Based on zip code level data.

^g^ Range, 0-30 mL/min/1.73 m^2^.

^h^ Range, 0.6-6.0 g/dL.

^i^ Range, 2-20 g/dL.

^j^ Range, 13-70.

We estimated dialysis facility market competition each year based on the facilities where prevalent patients living in each HSA received hemodialysis. The unit of observation (and initial outcome) in our negative binomial and predicted marginal effects models was hospital days per month.

### Additional Comparisons to Clarify Findings

Our primary aim was to determine whether acquisitions by dialysis chains were associated with changes in health outcomes at acquired facilities. Because facilities that were acquired had lower adjusted mortality and fewer hospital days before acquisitions compared with nonacquired facilities, findings from the DID models could be interpreted in several ways. We conducted analyses to examine 2 alternative explanations, focusing on our findings involving independently owned facilities.

First, we examined the possibility that DID estimates could be explained by changes implemented at nonacquired facilities in response to entry of a nearby dialysis chain. We did this by replacing nonacquired control facilities with facilities in HSAs where no acquisition occurred, which would not be expected to respond to acquisitions.

Second, we examined whether our study findings could be explained by a form of regression to the mean, which might occur if acquiring chains actively targeted facilities with healthier patients but could not fully distinguish systematic from random differences in patient health. If baseline differences in patient health among acquired vs nonacquired facilities were temporary and random, they would be expected to disappear with repeated sampling. We examined this possibility by comparing facility health outcomes in the preacquisition period with outcomes in the 2 years before entry into the study.

### Statistical Analysis

Because of the large sample size, we used a 10% standardized difference as a measure of significance when comparing patients in the preacquisition periods.^29^ We used a *P* value <.05 to evaluate the significance of changes over time in characteristics among comparison groups. When examining changes in unadjusted characteristics over time, we used linear and logistic models where each characteristic of interest is a function of case vs control before acquisition vs after acquisition and the interaction between case and after acquisition. In multivariable models, we obtained linear combinations of relevant coefficient estimates. We used the Δ method to transform regression results into estimated hospital days and multiplied predicted marginal effects by 12 to ascertain hospital days per patient-year. When analyzing hospitalizations, we used cluster-robust SEs to account for repeated observations within patients.^30^ eMethods in the Supplement provides more detail about the study design and covariate measurement.

## Results

### Baseline Characteristics

We identified 174 905 patients initiating dialysis between 2001 and 2015 (eFigure 1 in the Supplement). The 1875 acquired facilities comprised 36.5% of all facilities included in the study cohort, treating 84 991 patients (48.6%) initiating dialysis. There were 3259 nearby facilities (63.4% of facilities in the study cohort) that were not acquired, and 89 914 patients (51.4%) initiated dialysis at these facilities. Most acquisitions (63.5%) occurred between 2005 and 2007 (eTable 1 in the Supplement). Gambro (26 888 [31.6%]) independently owned facilities (20 346 [23.9%]), and Renal Care Group (16 161 [19.0%]) comprised 89.6% of all patients at acquired facilities. DaVita (33 879 [39.9%]), Fresenius (21 634 [25.5%]), and DSI/NRI (4731 [5.6%]) comprised 70.9% of all acquisitions. In the 3 years before acquisitions, 21 222 patients (22.4%) received dialysis at independently owned facilities, 11 319 (53.3%) of whom were at facilities acquired by dialysis chains. For-profit, independently owned facilities were more likely to be acquired than nonprofit facilities (eTable 2 in the Supplement). Among those at chain-owned facilities before acquisitions, 34 317 patients (46.7%) were at facilities acquired by other dialysis chains.

The mean (SD) age of the study population was 65 (15) years. The study population included 79 705 women (45.6%), with 24 409 (14.0%) of Hispanic ethnicity, 61 815 (35.3%) black, 105 272 (60.2%) white, and 1247 (0.7%) Native American. In the analysis of hospitalization days, median (interquartile range [IQR]) follow-up was 8 (4-12) months. A total of 7743 patients (4.4%) were lost to follow-up or changed dialysis modalities during the follow-up period, 19 899 patients (11.3%) were censored because they switched dialysis facilities, and 4616 patients (2.6%) were censored owing to loss of Medicare coverage. In the analysis of mortality, median (IQR) follow-up was 342 (135-365) days. A total of 6121 patients (2.1%) were censored because of kidney transplantation.

In the period before acquisitions, acquired facilities had more patients with coronary artery disease compared with nonacquired facilities. Acquired facilities had fewer patients who were black or Hispanic, and were more likely to be in areas with lower poverty rates and population densities. Following dialysis facility acquisitions, these differences in black populations, area-level poverty, and coronary artery disease narrowed (Table). We observed similar differences across acquisition groups when examining independent and chain-owned facilities (eTable 3 and eTable 4 in the Supplement).

### Mortality Analyses

Prior to acquisitions, the unadjusted 1-year probability of death was 19.6% at acquired facilities and 18.2% at nonacquired facilities. Following acquisitions, unadjusted mortality decreased 1.3 percentage points (from 19.6% to 18.3%; *P* < .001) at acquired facilities and 2.1 percentage points (18.2% to 16.1%; *P* < .001) at nonacquired facilities (eFigure 2 and eFigure 3 in the Supplement). In a multivariable Cox proportional hazards regression model including all facility-ownership types, mortality in the preacquisition period was nominally but not significantly lower at acquired vs nonacquired (reference) facilities (hazard ratio [HR], 0.97; 95% CI, 0.94-1.00). Adjusted mortality decreased 9.1% (95% CI, −12.0% to −6.2%) at acquired facilities vs 11.3% (95% CI, −14.2% to −8.4%) at nonacquired facilities. The DID was not statistically significant (*P* = .28).

Among facilities that were independently owned before acquisitions, unadjusted 1-year probability of death was 20.2% at acquired facilities and 19.5% at nonacquired facilities. Following acquisitions, unadjusted mortality decreased 1.1 percentage points (20.2% to 19.1%; *P* = .05) at acquired facilities and 3.8 percentage points (19.5% to 15.7%; *P* < .001) at nonacquired facilities. In a multivariable regression model stratified by facility ownership, adjusted mortality among independently owned facilities in the preacquisition period was lower at acquired vs nonacquired facilities (HR, 0.90; 95% CI, 0.84 to 0.95). Following acquisitions, adjusted mortality declined by 8.5% (95% CI, −2.5% to −14.1%) at independently owned acquired facilities vs 20.3% (95% CI, −25.8% to −14.3%) at independently owned nonacquired facilities. The DID was statistically significant (*P* = .005).

**Figure 2.**
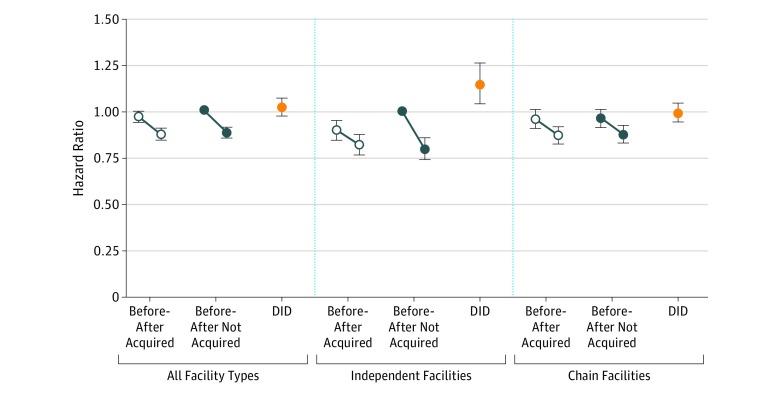
Adjusted Mortality Rates Before vs After Acquisitions, by Acquisition Assignment and Facility Ownership Hazard ratios for all facility types were obtained from 1 model, with not acquired and before acquisition serving as the reference. Hazard rates for independent and chain facilities were obtained from 1 model with interaction terms, where independent, not acquired and before acquisition served as reference. Before-after refers to the periods before and after acquisitions and 95% CIs were not adjusted for multiple comparisons. Facility ownership category is based on whether facilities were classified as independently owned or chain-owned in the period before acquisition by chains. DID indicates difference in differences; error bars, 95% CIs. eTable 7 in the Supplement provides the results of the full Cox proportional hazards regression models.

Among facilities that were chain-owned prior to acquisitions, the unadjusted 1-year probability of death was 19.4% at acquired facilities and 17.9% at nonacquired facilities. Following acquisitions, unadjusted mortality declined by 1.3 percentage points (19.4% to 18.1%; *P* < .001) at acquired facilities and 1.7 percentage points (17.9% to 16.2%; *P* < .001) at nonacquired facilities. In a multivariable regression model, adjusted mortality in the preacquisition period was not significantly different at acquired vs nonacquired facilities (HR, 1.00; 95% CI, 0.96 to 1.04). Following acquisitions, adjusted mortality declined by 9.4% (95% CI, −12.6% to −6.1%) at chain-owned acquired facilities vs 8.8% (95% CI, −12.1% to −5.5%) at chain-owned nonacquired facilities. The DID was not statistically significant (*P* = .81) (Figure 2; eTable 5 and eTable 7 in the Supplement).

### Hospitalization Analyses

In unadjusted analyses of all facility ownership types, acquired facilities had a mean of 17.2 (median, 5; IQR, 0-20) hospital days per patient-year before acquisitions, and nonacquired facilities had a mean of 19.0 days (median, 5; IQR, 0-22). Following acquisitions, unadjusted mean hospital days per patient-year declined by 0.8 (from 17.2 to 16.4; *P* < .001) at acquired facilities and 2.2 (19.0 to 16.8; *P* < .001) at nonacquired facilities (eFigure 4 in the Supplement). In a multivariable regression model including all facility ownership types, facilities that were acquired had a mean of 1.2 (95% CI, −1.7 to −0.8) fewer hospitalization days per patient-year than nonacquired facilities in the preacquisition period. Adjusted hospitalization rates declined following acquisitions in both groups, but this decline was less pronounced at acquired facilities; mean annual decline of 0.9 days per patient-year (95% CI, −1.3 to −0.5) at acquired facilities vs 2.0 days (95% CI, −2.5 to −1.6) at nonacquired facilities. The DID was statistically significant (*P* < .001).

In unadjusted analysis of facilities that were independently owned before acquisitions, acquired facilities had a mean of 17.1 (median, 5; IQR, 0-20) hospital days per patient-year, while nonacquired facilities had a mean of 21.2 (median, 6; IQR, 0-24) hospital days. Unadjusted mean hospital days at independently owned facilities did not decline significantly at acquired facilities and declined by 3.0 (from 21.2 to 18.2 days; *P* < .001) at nonacquired facilities. In a multivariable regression model stratified by facility ownership, independently owned acquired facilities had a mean of 2.9 (95% CI, −3.8 to −2.0) fewer hospitalization days per patient-year than nonacquired facilities in the preacquisition period. Adjusted mean hospitalization rates did not change following acquisitions at acquired facilities and declined following acquisitions by 2.6 days per patient-year (95% CI, −3.6 to −1.7 days) at nonacquired facilities. The DID estimate was statistically significant (*P* < .001).

**Figure 3.**
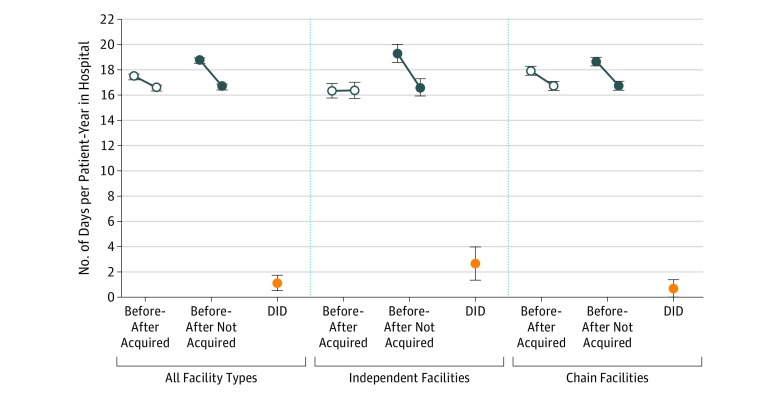
Predicted Number of Days per Patient-Year in the Hospital Before vs After Acquisitions by Acquisition Assignment and Facility Ownership Predicted probabilities are derived from the negative binomial regression models for each facility type illustrated in eTable 8 in the Supplement. The 95% CIs were obtained using the Δ method. Before-after refers to the period before and after acquisitions and 95% CIs were not adjusted for multiple comparisons. Facility ownership category is based on whether facilities were classified as independently owned or chain-owned in the period prior to acquisition by chains. DID indicates difference in differences; error bars, 95% CIs.

Among chain-owned facilities, unadjusted mean hospitalization days prior to acquisitions were 17.3 (median, 5; IQR, 0-20) at acquired facilities and 18.5 (median, 5; IQR, 0-21) at nonacquired facilities. Following acquisitions, unadjusted mean hospitalization rates declined 1.1 days (from 17.3 to 16.2; *P* < .001) at acquired facilities vs 2.0 days (from 18.5 to 16.5; *P* < .001) at nonacquired facilities. In a multivariable regression model, chain-owned acquired facilities had a mean of 0.7 (95% CI, −1.2 to −0.2) fewer hospitalization days than nonacquired facilities in the preacquisition period. Mean hospitalization rates declined by 1.2 (95% CI, −1.7 to −0.7) days at acquired facilities vs 1.9 (95% CI, −2.4 to −1.4) days at nonacquired facilities; *P* = .05 for DID estimate) (Figure 3; eTable 6 and eTable 8 in the Supplement).

### Additional Analyses

Our primary study findings, represented by the DID estimates among independently owned facilities, were not sensitive to alternative model specifications (eFigure 5 and eFigure 6 in the Supplement) and we did not find evidence that the estimated effect of acquisitions varied in the 3 postacquisition years or after ESRD payment reform in 2011 (eMethods in the Supplement).

In analyses where we replaced nonacquired nearby control facilities with nonacquired facilities in different HSAs, acquisitions of independently owned facilities continued to be associated with slower declines in mortality and hospitalization days, although the estimated DIDs effects were smaller. Mortality rates declined by 7.7% (95% CI, −13.4% to −1.8%) at acquired facilities vs 13.4% (95% CI −16.5% to −10.1%) at nonacquired facilities; P=.09 for DID estimate. Hospitalization rates increased by 0.5 days (95% CI, −0.7 to 1.8) at acquired facilities and declined by 2.0 days per patient-year (−2.6 to −1.4) at nonacquired facilities; *P* = .001 for DID estimate (eTable 9 in the Supplement). Differences in health outcomes in the preacquisition period persisted before entry into our study, making regression to the mean a less-likely alternative explanation to the study findings (eTable 10 in the Supplement).

## Discussion

In the 3 years before acquisitions by dialysis facility chains, acquired facilities had fewer adjusted hospitalization days and lower mortality rates compared with nonacquired facilities. Hospital days per patient-year and mortality declined over time and in the period following acquisitions in nearly all patient groups. However, these declines were slower or nonexistent among acquired facilities, enabling nonacquired facilities to catch up to nearby acquired facilities. Differences in the magnitude of decline in hospital days and mortality were most pronounced when examining facilities that were independently owned in the period before acquisition by a dialysis chain.

There are several possible reasons why acquisitions of independently owned facilities may have prevented patients at acquired facilities from realizing improvements in health outcomes that they would have otherwise experienced. Studies of chain acquisitions indicate that independent dialysis facilities with above-average costs and better-quality outcomes are more likely to be acquired by dialysis chains^31^ and that technical efficiency (defined as cost per dialysis treatment) at facilities may improve when large chains acquire inefficiently run independent facilities.^32^ It is possible that acquiring chains take advantage of opportunities for cost reduction at the expense of efforts to improve the quality of care delivered. Care disruptions associated with acquisitions could also affect health outcomes. For instance, changes in schedules, protocols, and other facility practices, as well as staff turnover following acquisitions, may temporarily disrupt patient care. Although we followed up facilities for only 3 years after acquisitions, the absence of significant temporal changes in the associations among acquisitions and health outcomes makes this explanation less likely. While opportunities for cost reduction may dictate decisions to acquire independent facilities, other considerations (such as the opportunity to increase purchasing power with private health insurers and suppliers) may factor more prominently in decisions to acquire other chains. Differences in strategic considerations could explain why acquisitions of one chain by another chain were less closely associated with health outcomes.

Previous studies have found that health care clinicians may respond to nearby competitive changes in local markets.^33,34^ In our study, this competitive pressure or spillover of quality-enhancing practices from acquiring chains to nearby facilities could have led nearby nonacquired facilities to improve care delivery following chain acquisitions. When we examined this possibility in an additional analysis, we found that the magnitudes of estimated acquisition outcomes were smaller when comparing acquired facilities with nonacquired facilities that were not in the same HSA, which is consistent with possible competitive spillover. However, this phenomenon did not fully explain the estimated outcome of acquisitions, as acquisitions also appear to be more directly associated with health outcomes at acquired facilities.

### Limitations

This study has several limitations. Because we focused on patients initiating dialysis, we relied on comorbidity data from the CMS-2728, which can have limited detection of some comorbidities.^35^ We only stratified acquisitions by independent vs chain status in the preacquisition period and did not formally examine other potential facility characteristics that might modify the association between acquisitions and patient health outcomes, including facility practices that may have influenced changes in health outcomes and in the numbers of patients treated. We did not examine whether acquisitions influenced health outcomes at other facilities owned by acquiring chains. Because baseline differences in health outcomes in the period before acquisitions persisted, despite adjustment for characteristics available to us, we were unable to use matching techniques to eliminate baseline differences. In addition, our findings may be confounded by differences among facilities in the underlying health and/or reporting of comorbidities following acquisitions. Although we cannot exclude this possibility, changes in patient characteristics associated with acquisitions were small, suggesting that unobserved differences were small (eTable 11 in the Supplement).

## Conclusions

Mergers and acquisitions are increasingly common in health care,^20,36^ and consolidation in the dialysis industry through facility mergers and acquisitions continues at a steady pace.^37,38^ Declines in dialysis market competition have been associated with more frequent hospitalization rates.^12^ Findings from this study suggest that dialysis facility acquisitions may be associated with impaired quality of care delivered at acquired facilities through mechanisms separate from market competition. These results highlight the need for critical evaluation of commonly used arguments that horizontal integration among dialysis facilities and among other health care organizations yield meaningful improvements in the quality of care. Although dialysis facility acquisitions may result in financial and operational efficiencies, declines in health outcomes should be avoided.

## Notes

Supplement. **eMethods.** Detailed Methodology **eTable 1.** Number of Acquired Facilities and Nearby Non-acquired Facilities by Year **eTable 2.** Dialysis Facility Characteristics Prior to Acquisitions **eTable 3.** Baseline Characteristics Among Independently Owned Facilities **eTable 4.** Baseline Characteristics among Chain-Owned Facilities **eTable 5.** Summary of Cox Regression Results Examining Mortality **eTable 6.** Summary of Regression Results Examining Hospital Days per Patient-Year **eTable 7.** Full Results from Cox Models Examining Association Between Acquisitions and Mortality **eTable 8.** Negative Binomial Models Examining Association Between Acquisitions and Hospital Days **eTable 9.** Estimates from Models Using Alternative Controls **eTable 10.** Examining for “Regression to the Mean” **eTable 11.** Assessing Differences in Comorbidities Among Comparison Groups **eFigure 1.** Study Selection Diagram **eFigure 2.** Unadjusted One-Year Probabilities of Death **eFigure 3.** Percentage Point Improvements in Unadjusted Mortality Rates in the Period After Acquisitions **eFigure 4.** Unadjusted Hospital Days per Patient-Year **eFigure 5.** Sensitivity Analyses Focusing on Mortality **eFigure 6.** Sensitivity Analyses Focusing on Hospital Days per Patient-Year
