# Supplementary material for: Association of Hospitalization and Mortality Among Patients Initiating Dialysis With Hemodialysis Facility Ownership and Acquisitions
Source: JAMA Netw Open. 2019 May 17;2(5):e193987. doi: 10.1001/jamanetworkopen.2019.3987 (PMC6537810; doi:10.1001/jamanetworkopen.2019.3987)
Supplement: Supplement. — eMethods. Detailed Methodology eTable 1. Number of Acquired Facilities and Nearby Non-acquired Facilities by Year eTable 2. Dialysis Facility Characteristics Prior to Acquisitions eTable 3. Baseline Characteristics Among Independently Owned Facilities eTable 4. Baseline Characteristics among Chain-Owned Facilities eTable 5. Summary of Cox Regression Results Examining Mortality eTable 6. Summary of Regression Results Examining Hospital Days per Patient-Year eTable 7. Full Results from Cox Models Examining Association Between Acquisitions and Mortality eTable 8. Negative Binomial Models Examining Association Between Acquisitions and Hospital Days eTable 9. Estimates from Models Using Alternative Controls eTable 10. Examining for “Regression to the Mean” eTable 11. Assessing Differences in Comorbidities Among Comparison Groups eFigure 1. Study Selection Diagram eFigure 2. Unadjusted One-Year Probabilities of Death eFigure 3. Percentage Point Improvements in Unadjusted Mortality Rates in the Period After Acquisitions eFigure 4. Unadjusted Hospital Days per Patient-Year eFigure 5. Sensitivity Analyses Focusing on Mortality eFigure 6. Sensitivity Analyses Focusing on Hospital Days per Patient-Year [file jamanetwopen-2-e193987-s001.pdf]

Supplementary Online Content

Erickson KF, Zhao B, Niu J, et al. Association of hospitalization and mortality among patients initiating dialysis with hemodialysis facility ownership and acquisitions. *JAMA Netw Open*. 2019;2(5):e193987. doi:10.1001/jamanetworkopen.2019.3987

eMethods. Detailed Methodology
eTable 1. Number of Acquired Facilities and Nearby Non-acquired Facilities by Year
eTable 2. Dialysis Facility Characteristics Prior to Acquisitions
eTable 3. Baseline Characteristics Among Independently Owned Facilities
eTable 4. Baseline Characteristics among Chain-Owned Facilities
eTable 5. Summary of Cox Regression Results Examining Mortality
eTable 6. Summary of Regression Results Examining Hospital Days per Patient-Year
eTable 7. Full Results from Cox Models Examining Association Between Acquisitions and Mortality
eTable 8. Negative Binomial Models Examining Association Between Acquisitions and Hospital Days
eTable 9. Estimates from Models Using Alternative Controls
eTable 10. Examining for "Regression to the Mean"
eTable 11. Assessing Differences in Comorbidities Among Comparison Groups
eFigure 1. Study Selection Diagram
eFigure 2. Unadjusted One-Year Probabilities of Death
eFigure 3. Percentage Point Improvements in Unadjusted Mortality Rates in the Period After Acquisitions
eFigure 4. Unadjusted Hospital Days per Patient-Year
eFigure 5. Sensitivity Analyses Focusing on Mortality
eFigure 6. Sensitivity Analyses Focusing on Hospital Days per Patient-Year

This supplementary material has been provided by the authors to give readers additional information about their work.

© 2019 Erickson KF et al. *JAMA Network Open.*

eMethods. Detailed Methodology

1) *Determining Facility Ownership and Acquisitions:*

We used two data sources to identify dialysis facility owners: Annual dialysis facility surveys included in the United States Renal Data System (USRDS) database, and data from Dialysis Facility Compare (DFC). When obtaining data from DFC, we used ownership from the report closest in time to January 1st of the calendar year of interest. When we examined changes in ownership following two large dialysis facility acquisitions, we noticed that the Annual Facility Surveys appeared more accurate. Notably, a small number of facilities that were acquired continued to report the previous owner in DFC through as late as 2011. We suspect that this is because DFC relies on facilities reporting ownership changes to their regional end-stage renal disease (ESRD) Network, while the Centers for Medicare and Medicaid Services (CMS) mandates that each facility complete the survey annually.

Because the facility survey and DFC both contained information about large dialysis facility chains, we used the more up-to-date data contained in the Annual Facility Surveys to assign ownership to facilities when a facility was noted to be owned by a large chain in the Facility Survey. However, the Facility Surveys did not contain information about smaller regional chains. In instances where the Facility Survey did not report a facility as being owned by a large chain, but where DFC assigned a facility to a regional chain, we used the DFC data to assign ownership. Only in instances where both the Facility Report and DFC listed a facility as being independent did we consider it as such.

We defined facility ownership change when we observed a change in chain-ownership from one calendar year to the next. In order for a facility to be included in our analysis, we also required that it have the same owner in the 3-years prior to and in the 3-years following the reported ownership change. Out of 2,270 ownership changes that we identified during the study period, 391 (17%) were excluded due to subsequent ownership changes occurring within 3-years. Because we were interested in acquisitions by dialysis facility chains, we excluded facilities in the rare instance where a facility changed from chain-ownership to independent ownership.

2) *Difference-in-Differences Model:*

Un-stratified analyses

The difference-in-differences models estimated whether the change in hospital days or hazard of death among patient starting dialysis before *versus* after acquisitions depended upon whether or not patients were at facilities directly affected by acquisitions.

Below is the equation used in our primary un-stratified analysis examining hazard of death:

Equation 1:

$$Hazard\ of\ death\ (t) = \lambda_0(t) * e^{[\alpha(Affected)+\ \beta(post-acquisition)+\gamma(PostAcquisition*Affected))+\ \delta 1(AcqYr2005)+\ \delta 2\ (AcqYr2006)\ldots \delta 11\ (AcqYr2013)+\ \mu(X)]}$$

- *"Affected"* is a binary variable that denotes whether a patients initiated dialysis at a facility that was acquired.
- *"PostAcquisition"* is a binary variable denoting whether a patient initiated dialysis in the post-acquisition period. The actual calendar years that define the *PostAcquisition* period differ depending on the acquisition to which a patient is assigned.
- *"AcqYr2005"* through *"AcqYr2013"* are dummy variables (with cohort "*AcqYr2004*" as the referent) representing the acquisition cohort to which a patient has been assigned.
- *"X"* is a vector of all model covariates.

Within this model framework, regression coefficient exp(γ) represents an estimate of the effect of acquisitions on the hazard of death. Results from this model can also be used to estimate the change in adjusted mortality following acquisition separately for patients at acquired and non-acquired facilities. For patients at non-acquired facilities, the coefficient *exp(β)* characterizes the estimated change in mortality following acquisitions. For patients at acquired facilities, the combination of *exp(β + γ)* characterizes the estimated change in mortality following acquisitions.

© 2019 Erickson KF et al. *JAMA Network Open.*

The negative binomial models of hospital days had a similar design, except that there were also covariates for each 30-day interval since starting dialysis and multiple records for each patient. We converted model estimates into average marginal effects, and multiplied days-per-month by 12 to obtain days-per-patient-year.

Stratified analyses

When stratifying by facility ownership type, we supplemented the model described in **Equation 1** with the following additional regression terms with corresponding coefficients to separately estimate acquisition effects at independent *versus* chain-owned facilities:

1) A term identifying chain-owned facilities prior to acquisitions 'η'
2) An interaction between a facility that is acquired and chain ownership prior to acquisitions: 'ρ'
3) An interaction between chain-ownership prior to acquisitions and initiation of dialysis in the period following acquisitions: 'τ'
4) An interaction between chain-ownership prior to acquisitions, initiation of dialysis in the period following acquisitions, and being at a facility that was acquired by a chain: 'θ'

In this stratified model, the coefficients α, β, and γ can be interpreted as coefficients specific to independently-owned facilities. The additional regression terms describe how temporal changes in outcomes differed among patients at facilities that were owned by chains prior to acquisitions. In particular:

- The regression coefficient $exp(\theta)$ describes the extent to which the difference-in-difference estimate varies between patients at chain-owned facilities and those not at chain-owned facilities prior to acquisitions.
- The combination of $exp(\beta + \tau)$ describes how mortality changed in the period following acquisitions among patients at facilities that were owned by chains prior to acquisitions and that were not acquired.
- The combination of $\exp(\beta + \tau + \gamma + \theta)$ describes how mortality changed in the period following acquisitions among patients at facilities that were owned by chains prior to acquisitions and that were acquired by a separate chain.
- The combination of $\exp(\gamma + \theta)$ represents a difference-in-difference estimate specific to patients at facilities that were owned by chains in the period prior to acquisitions.

3) *Examining Early Facility Switches:*

Our previous observation that patients are more likely to switch dialysis facilities soon after developing ESRD informed our decision to assign patients to a facility based on where they received dialysis at the start of their fourth dialysis month. However, we are unaware of specific sources where this phenomenon has been published. Consequently, we conducted an analysis to verify this assertion.

Among patients initiating in-center hemodialysis between 2001 and 2015, we examined the likelihood of changing dialysis facilities in each 90-day interval during the first 360 days of dialysis. Each probability was conditional upon receiving in-center hemodialysis at the start of the 90-day interval. In the first 90 days of dialysis, 21.5% of patients switched dialysis facilities at least once. This declined to 9.2%, 8.2%, and 7.6% among patients in the 2nd, 3rd, and 4th, 90-day intervals, respectively, confirming that facility switches are more common in the first 90 days of dialysis.

4) *Measuring Market Competition:*

We calculated a commonly-used metric of market competition – the Herfindahl-Hirschman Index (HHI) – based on where patients lived and the facilities where they received dialysis. We calculated this metric separately for each calendar year using a point-prevalent cohort of all patients receiving in-center hemodialysis in the United States on the first day of the year. In our primary models, we defined geographic markets using hospital service areas (HSAs), although we varied this definition in sensitivity analyses. The equation used to calculate Herfindahl-Hirschman Index (HHI) is as follows:

$$HHI_{hospital\ service\ area} = \sum_{i=1}^{n} s_i^2$$

Where S_i represents the proportion of patients living in an HSA receiving dialysis at the ith firm in the HSA.
We calculate a measure of "observed HHI" for each hospital service area (HSA) from the following three steps:

1. Calculate a "first-stage" competition measure for each HSA (using the equation above), based on sum of squared market shares of firms where patients living in each HSA choose to dialyze. In this stage all patients residing in a given HSA define the "market" for each firm-HSA pair. Firms do not have to be located in the same HSA where patients reside, and a given dialysis facility can be included in the calculation of HHI for multiple HSAs if patients from multiple HSAs dialyze at that facility. For the purposes of this step, facilities owned by the same dialysis chain were considered to be one firm. The market share for a firm in an HSA is equal to the proportion of patients in that HSA who choose to dialyze at that firm. For example, in an HSA where half of the patients receiving dialysis went to one of four facilities owned by one firm and the other half of patients went to one of two facilities owned by a second firm, the market share would be considered to be split evenly across the two firms, with an HHI for that HSA of $0.5^2 + 0.5^2 = 0.5$.

2. Calculate a dialysis-facility-level measure of competition, using a weighted average of the "first-stage" HSA-level HHIs for patients who actually dialyze at each facility. This measure is calculated for each separate facility, regardless of which firm owns a facility. It assumes that facilities compete for patients within HSAs and can discriminate against patients living in different HSAs when competing against rival firms.

3. Calculate a "second-stage" HSA-level measure of competition from a weighted average of the facility-level-HHIs at facilities where patients residing in each HSA receive dialysis.

In summary, this index represents a weighted average of competition indices for facilities that treat patients in a given HSA, where facility competition indexes are, in turn, weighted by choices available to patients they treat.

5) Assigning Patients to Acquisition Cohorts:

In several instances, patients appeared in multiple acquisition cohorts. This could occur, for example, if a patient started dialysis at a facility (we will call this **Facility A**) that was in the same hospital service area as a facility that had been acquired (we will call this **Facility B**). Suppose that **Facility B** was acquired between 2004 and 2005, and a patient started dialysis at nearby **Facility A** in 2006. If **Facility A** maintained the same owner through 2007, the patient would be included in the post-acquisition, "control" group for the acquisition involving **Facility B** in the 2004-2005 acquisition cohort. However, if **Facility A** then changed owners between 2008 and 2009, this same patient would be included in the pre-acquisition group for patients at the acquired **Facility A** in the 2008-2009 acquisition cohort. This is one of several scenarios where a patient could appear more than once after combining all seven acquisition cohorts.

In order to ensure that each patient only appeared once in our analysis cohort, we used the following set of rules to assign patients to one and only one acquisition cohort:

1) If a patient was in the acquisition group of one cohort and the "control" group of another cohort, we assigned them to the cohort where they appeared in the acquisition group.
2) If a patient was in two acquisition groups or two control groups, we randomly assigned them to one of the two groups.

In sensitivity analyses, we included all patients under the assumption that they represented independent observations. Notably, our findings were not substantially different in this sensitivity analysis.

6) Examination of Proportional Hazards Assumption:

We examined our stratified Cox model for violation of the proportional hazards assumption by separately examining a "complete-case" sample and a "full-cohort" sample, In the "full-cohort" sample, we excluded variables that were multiply imputed. In both samples, we tested the null hypothesis that the slope for the scaled Schoenfeld residual on time for each model covariate was zero, and the global hypothesis that all slopes were zero.

Based on this test, the following covariates violated the proportional hazards assumption using a p-value of <0.01 as a measure of significance: immobility, Medicare coverage, employer-based group coverage, serum albumin <2, serum albumin 2-3. However, when we visualized the log-log survival curves involving these variables, there was no clear evidence of a change in the hazard rate ratio over time.

© 2019 Erickson KF et al. *JAMA Network Open.*

7) Replacing Nearby Non-Acquired Facilities with Alternative "Controls":

An alternative interpretation of our study findings, when considering the baseline differences in health outcomes among acquired *versus* non-acquired independently-owned facilities, is that acquisitions led to a narrowing in health outcomes because of responses initiated by nearby non-acquired facilities. To examine this possibility, we created a study cohort using an alternative "control" group. Specifically, we replaced our comparison groups of unaffected patients with patients initiating dialysis in hospital service areas where no-acquisition occurred. Facilities in this alternative control group would not be expected to respond to acquisitions, since there were no nearby acquisitions to encourage changes in practices. Using this alternative cohort, we ran regression models identical to those described in our primary analyses. We focus our discussion on findings related to independently-owned facilities.

8) Examining for "Regression to the Mean":

We examined the possibility that slower declines over time in hospital days (and mortality) among acquired *versus* non-acquired facilities can be explained by "regression to the mean". If this alternative interpretation of our primary study findings were true, and observed differences in outcomes in the pre-acquisition period were simply due to chance, we would expect to observe a narrowing of pre-acquisition differences in outcomes in the years before and after the baseline (i.e. pre-acquisition) period. Repeated sampling of patients initiating dialysis before enrollment in our study would be expected to approach population means in the same way as repeated sampling after acquisitions.

To examine this possibility, we identified patients starting dialysis at facilities included in our primary study cohort in the 2-years prior to entry into the cohort (i.e. 4th and 5th years prior to acquisitions). This required expanding our cohort to patients initiating dialysis in 1999 and 2000. We identified patients initiating dialysis in the 2-years prior to entry into the study for all 10 acquisition cohorts and included them in our primary regression models. To simplify the creation of this expanded cohort, and to ensure that adding these data would not lead us to exclude patients from our primary cohort, we deviated from our primary study selection approach in several ways when adding the two prior patient-years:

1) We did not require that facilities had "stable" ownership in the 4th and 5th years prior to inclusion in our study cohort. This ensured that there would not be a change in facilities categorized as acquired and non-acquired in this analysis.
2) Patients could appear more than once if the 2nd appearance occurred in the newly-added years.

We added the following regression coefficients to the stratified difference-in-difference models: 1) dialysis initiation "before baseline" (i.e. 4 or 5 years prior to acquisitions); 2) interaction between "acquired facilities" and dialysis initiation "before baseline"; 3) interaction between "chain-ownership" and dialysis initiation "before baseline, and; 4) three-way interaction between "before baseline", "chain-ownership" and "acquired facilities".

Within this model, the estimated effect of the *(acquired facilities)* (before baseline)* interaction characterizes whether differences in health outcomes among independently-owned acquired *versus* non-acquired facilities narrowed in the before-baseline period. The three-way interaction *(before baseline)*(chain-ownership)*(acquired facilities)* indicates whether narrowing (or widening) of the outcomes gap in the period before baseline differed at chain *versus* independent facilities.

Because our study findings were most robust when examining independent facilities, we focused our discussion on differences between acquired and non-acquired facilities that were independently-owned in the pre-acquisition period. However, we report results involving both independent and chain-owned facilities.

9) Assessing Differences in Comorbidities among Comparison Groups:

It is not obvious how differences in reported patient characteristics over time at independently-owned facilities and chain-owned facilities would be expected to influence the measurement of health outcomes. To examine this issue, we used estimates from our stratified regression models to compare predicted probabilities hospitalization days and death among patient subgroups. In order to retain all patients used in the primary analyses, and to avoid unnecessarily complicating the analyses with multiple imputation, we excluded parameters with high numbers of

© 2019 Erickson KF et al. *JAMA Network Open.*

missing values: laboratory values, BMI, and eGFR. When modeling the probability of death, we assumed a parametric survival function with an exponential distribution (unlike all other analyses, which used Cox regression).

10) *Sensitivity Analyses*:

We tested the sensitivity of our stratified analyses to a number of alternative model specifications. We focus our discussion of these sensitivity analyses on regression coefficients pertaining to independently-owned facilities but also include a coefficient identifying whether the difference-in-differences estimate varied among independently-owned *versus* chain-owned facilities **(eFigures5-6)**. In all sensitivity analyses, we used the full regression model described in **Equation 1** and supplemented with additional covariates for stratified analyses.

A) We conducted sensitivity analyses where we included dummy variables representing the calendar year of dialysis initiation as additional model covariates.
B) It is possible that hospital service areas do not accurately represent a local market for dialysis facilities. To examine this potential inaccuracy, we adapted our method of calculating HHI to calculate a market concentration index at the zip-code level. This involved substituting the hospital service area (HSA) of patient residences with their zip-codes in steps 1 and 3 of the algorithm described above in *"measuring market competition."* Within each HSA, we then compared zip-code level HHIs among acquired and non-acquired facilities both before and after acquisitions. We did this using the following linear regression model with fixed-HSA effects:

Equation 2.

$$HHI_{zipcode} = \alpha + \beta * Acquired + \gamma * PostAcquisition + \delta * (Acquired) * (PostAcquisition) + \varepsilon$$

In this analysis, we found small but statistically-significant differences in the change in zip-code level HHIs (ranging from 0 to 0.02) among acquired and nearby non-acquired facilities following acquisitions. Because of this slight variation, we performed an additional sensitivity analysis where we controlled for market competition prior to and following acquisitions based on patient zip-codes.

C) We also conducted a sensitivity analysis where we used counties, rather than HSAs to define markets. This involved calculating county-level market competition indices in steps 1 and 3 described in "*measuring market competition."* In this analysis we also defined geographic proximity of facilities according to counties (rather than HSAs), meaning that the control group consisted of non-acquired facilities in the same *county* as acquired facilities.
D) We conducted a sensitivity analysis where we excluded Gambro facilities that were divested in the process of DaVita's acquisition of Gambro and Renal Care Group (RCG) facilities that were divested in the process of Fresenius's acquisition of RCG. We identified these facilities in instances where they changed ownership at the time of DaVita's acquisition of Gambro to a chain other than DaVita or where they changed ownership at the time of Fresenius's acquisition of RCG to a chain other than RCG.
E) We conducted sensitivity analyses where we excluded patient comorbidities where reporting on the Medical Evidence Report may be less certain. Specifically, we excluded cancer, heart failure, cerebrovascular disease, and coronary disease.
F) We conducted a sensitivity analysis where we only considered facilities to have been acquired if the acquisition was by a large dialysis organizations (LDOs). We used the following definition of LDO adopted by the Centers for Medicare and Medicaid services: an organization with at least 200 facilities in a given year.
G) We conducted a sensitivity analysis where we did not exclude patients starting dialysis in the 6-months prior to and following a dialysis acquisition. In this analysis, we assumed that the post-acquisition periods began on January 1st in the year of ownership change.
H) We conducted a sensitivity analysis where we did not require that patients only appear in the combined cohort one time. We did this by assuming that each patient contributed an independent observation, irrespective of the number of times that their record appeared in our combined cohort.

© 2019 Erickson KF et al. *JAMA Network Open.*

I) When examining mortality, we performed an additional analysis where we included all patients who started dialysis and who lived to the first day of the fourth dialysis month. This "expanded cohort" included patients who did not qualify for Medicare after 3 months of dialysis.

J) When examining hospital days, we performed two additional sensitivity analyses where we included: 1) hospital service area fixed effects, and; 2) dialysis facility fixed effects. We used linear probability models rather than negative binomial models in order to facilitate computation.

K) We also examined whether the change in hospital days and mortality from acquisitions at independently-owned facilities differed in the years following the 2011 end-stage renal disease (ESRD) payment reform. We did this in a model that only included patients at independently-owned facilities. In this model, we tested for a three-way interaction between the difference-in-difference estimate – (after acquisitions) * (at an acquired facility) * (initiating of dialysis on or after 2011).

L) We examine whether our analysis of mortality was sensitive to inclusion of patients at the start of dialysis. To do this we assigned patients to the facility where they initiated dialysis. Unlike our primary analyses, we did not exclude patients who died in the first 90 days of ESRD. We only conducted this sensitivity analysis for mortality, since hospitalizations required Medicare claims, which >40% of patients in our cohort did not have until after 90 days of ESRD.

We examined for a potential time-varying effect of acquisitions on mortality and hospitalizations among patients at independent facilities. This was done within the Cox and negative binomial models by creating binary indicator variables representing each of the three years following acquisitions, and by interacting these variables with an indicator of whether or not a patient was at a facility that had recently been acquired. These interaction terms identified potential variation in the acquisition effect over time. By testing the joint hypotheses that the *"(post-acquisition year)*(acquisition group)"* interaction terms were zero, we were able to assess whether the effect of acquisitions varied over time. In these analyses, we did not find evidence that the associations between acquisitions and each outcome varied over time in the post-acquisition period. In the case of mortality, the p-value for the test for heterogeneity in interaction terms was 0.9. In the case of hospitalization days, the p-value for the test of heterogeneity in interaction terms was 0.7.

eTable1. Number of Acquired Facilities and Nearby Non-acquired Facilities by Year

Year of acquisition	2004	2005	2006	2007	2008	2009	2010	2011	2012	2013	Total
Acquired	113	331	483	377	58	88	79	85	203	58	1,875
Nearby not-acquired*	226	327	586	401	147	330	206	194	568	274	3,259

*Not-acquired facilities are in the same hospital service area as acquired facilities. All facilities listed above had the same owner in the three years before and after the acquisition year.

eTable2. Dialysis Facility Characteristics Prior to Acquisitions

	Independently-owned		Chain-owned	
	Not acquired	Acquired	Not acquired	Acquired
For-profit facility*, %	51.71	80.11	90.05	96.26
Size, mean (sd)	118 (65)	96 (58)	102 (57)	94 (51)
Registered nurses per 100 patients, mean (sd)	11 (28)	7 (21)	5 (4)	6 (7)
Social workers per 100 patients, mean (sd)	1 (2)	1 (1)	1 (1)	1 (1)
Techs per 100 patients, mean (sd)	3 (4)	3 (4)	2 (3)	2 (3)
Stations per 100 patient, mean (sd)	23 (16)	26 (45)	26 (15)	26 (12)
Offers night shift, %	0	0	0	0.14
Offers home dialysis, %	50.08	56.02	46.76	48.80

Note: Characteristics are weighted by the proportion of patients at each facility type. 289 patients were excluded due to no information about for-profit status of their dialysis facility. We examined the dialysis facility chain sizes in 2015. Out of 97 dialysis chains reported 2015, 88 had fewer than 50 facilities and 6 had between 50 and 200 facilities.

© 2019 Erickson KF et al. *JAMA Network Open.*

eTable 3. Baseline Characteristics among Independently-owned Facilities

	Before Acquisition		After Acquisition		P value of interaction
	Acquired (n=11,319)	Not Acquired (n=9,903)	Acquired (n=9,027)	Not Acquired (n=7,950)	
Demographic and socioeconomic					
Age group					
18-49 -% *	13.3	18.1	13.9	18.9	0.98
50-64 -%	23.0	27.3	24.5	28.5	0.64
65-74 -%	28.9	26.2	27.7	24.0	0.20
more than 75 -% *	34.8	28.5	33.8	28.7	0.24
Female -%	46.5	46.2	46.4	44.5	0.12
Race					
White -% *	71.3	51.4	71.2	52.1	0.40
Black -% *	24.8	42.0	25.5	40.5	0.03
Native American -%	0.9	0.6	0.8	0.6	0.64
"Other" -% *	2.9	6.0	2.5	6.8	0.005
Hispanic ethnicity -% *	9.6	16.4	9.5	17.1	0.30
Medicaid eligible -% *	33.6	42.1	34.1	45.4	0.01
Uninsured -% *	6.7	11.0	7.5	11.6	0.41
Zip code median household income ($10K) -Median(IQR) *±	4.7 (2.1)	4.3 (2.3)	4.7 (2.2)	4.3 (2.3)	0.15
Zip code poverty rate per 100 residents -Median(IQR) *±	15.4 (13.3)	19.5 (16.1)	15.1 (12.8)	19.4 (15.7)	0.07
Health					
Cancer -%	7.8	6.4	8.2	7.5	0.12
Heart failure -%	39.2	34.9	37.4	34.5	0.19
Cerebrovascular disease -%	11.8	11.0	11.0	11.6	0.03
Diabetes -%	54.1	53.4	55.9	55.4	0.88
Coronary disease -% *	33.5	27.2	27.0	24.2	<.001
Drug or alcohol abuse -%	1.9	3.0	2.1	3.0	0.34
Immobility -%	6.1	8.1	7.9	10.2	0.78
eGFR (ml/min/1.73m2) -Median(IQR) *	8.4 (5.6)	7.5 (5.0)	8.9 (5.6)	8.1 (5.4)	0.19
Serum albumin level (g/dL) -Median(IQR)	3.2 (0.9)	3.2 (0.9)	3.2 (0.9)	3.2 (1.0)	0.05
Hemoglobin (g/dL) -Median(IQR) *	10.0 (2.2)	9.7 (2.1)	9.9 (2.1)	9.5 (2.1)	0.33
Body Mass Index (kg/m2) -Median(IQR)	26.6 (8.4)	26.1 (8.7)	27.4 (9.4)	26.6 (8.9)	0.009
Population Density					
Metropolitan -% *	80.6	96.0	79.9	96.4	0.10
Micropolitan -% *	8.2	2.1	9.1	1.9	0.07
Rural & small-town -% *	11.2	1.9	11.0	1.7	0.55
HHI -Median(IQR) *	0.4 (0.3)	0.3 (0.2)	0.5 (0.3)	0.3 (0.3)	<.001
Distance between home and facility -Median(IQR)	4.7 (9.0)	3.6 (5.5)	4.3 (8.7)	3.5 (5.4)	0.28

IQR is inter-quartile ratio. *Indicates >10% standardized difference in characteristics in the pre-acquisition period. †represents the statistical significance of the interaction term in a model where each characteristic of interest is a function of case vs. control, pre-acquisition vs. post-acquisition, and the interaction between case and post-acquisition. ± based on zip-code level data. Logistic and linear regression was used for binary and continuous outcomes, respectively.

© 2019 Erickson KF et al. *JAMA Network Open.*

eTable 4. Baseline Characteristics among Chain-owned Facilities

	Before Acquisition		After Acquisition		P value of interaction
	Acquired (n=34,317)	Not Acquired (n=39,204)	Acquired (n=30,328)	Not Acquired (n=32,857)	
Demographic and socioeconomic					
Age group					
18-49 -%	15.8	18.1	15.8	18.2	0.96
50-64 -%	25.2	27.9	27.4	29.4	0.09
65-74 -%	27.8	26.6	26.8	25.9	0.75
more than 75 -%	31.2	27.3	30.0	26.6	0.30
Female -%	46.0	46.3	44.9	44.4	0.12
Race					
White -% *	63.4	55.8	63.9	56.4	0.98
Black -% *	31.8	40.4	31.6	39.5	0.21
Native American -%	1.2	0.4	1.0	0.3	0.32
"Other" -%	3.6	3.4	3.5	3.8	0.004
Hispanic ethnicity -% *	11.4	16.9	11.7	16.4	0.02
Medicaid eligible -%	39.0	41.4	40.0	42.4	0.96
Uninsured -%	9.0	12.0	9.3	11.6	0.04
Zip code median household income ($10K) -Median(IQR) ±	4.4 (2.2)	4.3 (2.5)	4.4 (2.2)	4.4 (2.5)	0.01
Zip code poverty rate per 100 residents -Median(IQR) *±	17.2 (14.6)	19.2 (17.6)	17.1 (14.4)	18.3 (17.3)	0.00
Health					
Cancer -%	6.4	6.1	7.5	6.3	0.003
Heart failure -%	34.8	32.3	34.7	31.1	0.03
Cerebrovascular disease -%	10.1	9.4	10.2	9.3	0.66
Diabetes -%	55.1	55.2	55.4	56.7	0.03
Coronary disease -% *	26.8	21.2	20.8	16.4	0.58
Drug or alcohol abuse -%	2.0	2.4	2.4	2.6	0.24
Immobility -%	5.1	5.5	6.7	7.2	0.87
eGFR (ml/min/1.73m2) -Median(IQR)	8.1 (5.3)	7.9 (5.4)	8.6 (5.6)	8.3 (5.5)	0.05
Serum albumin level (g/dL) -Median(IQR)	3.2 (0.9)	3.1 (0.9)	3.2 (0.9)	3.1 (1.0)	0.05
Hemoglobin (g/dL) -Median(IQR)	10.0 (2.1)	9.8 (2.2)	9.9 (2.0)	9.5 (2.1)	<.001
Body Mass Index (kg/m2) -Median(IQR)	26.6 (8.7)	26.8 (9.0)	27.4 (9.5)	27.5 (9.5)	0.001
Population Density					
Metropolitan -% *	79.9	95.6	79.5	95.6	0.63
Micropolitan -% *	11.9	2.2	12.3	2.4	0.16
Rural & small-town -% *	8.2	2.2	8.3	2.0	0.05
HHI -Median(IQR)*	0.5 (0.3)	0.4 (0.1)	0.5 (0.3)	0.4 (0.1)	0.52
Distance between home and facility -Median(IQR)	4.2 (8.3)	4.0 (5.3)	4.2 (8.8)	3.9 (5.3)	0.005

IQR is inter-quartile ratio. *Indicates >10% standardized difference in characteristics in the pre-acquisition period. †represents the statistical significance of the interaction term in a model where each characteristic of interest is a function of case vs. control, pre-acquisition vs. post-acquisition, and the interaction between case and post-acquisition. ± based on zip-code level data. Logistic and linear regression was used for binary and continuous outcomes, respectively.

© 2019 Erickson KF et al. *JAMA Network Open.*

eTable 5. Summary of Cox Regression Results Examining Mortality

**Un-Stratified Analysis (All Facility Ownership Types)**				
	HR	LCI	UCI	p-value
acquired vs. non-acquired facility	0.97	0.94	1.00	0.067
change following acquisitions (non-acquired facility)	0.89	0.86	0.92	0.000
change following acquisitions (acquired facility)	0.91	0.88	0.94	0.000
difference in change following acquisitions	1.02	0.98	1.07	0.281
**Stratified Analysis**				
	HR	LCI	UCI	p-value
Independently-owned Facilities				
acquired vs. non-acquired facility, exp(α)	0.90	0.84	0.95	0.001
change following acquisitions (non-acquired facility), exp(β)	0.80	0.74	0.86	0.000
change following acquisitions (acquired facility), exp($\beta+\gamma$)	0.92	0.86	0.97	0.006
difference in change following acquisitions, exp(γ)	1.15	1.04	1.26	0.005
Chain-owned Facilities				
	HR	LCI	UCI	p-value
acquired vs. non-acquired facility, exp($\alpha+\rho$)	1.00	0.96	1.04	0.960
change following acquisitions (non-acquired facility) , exp($\beta + \tau$)	0.91	0.88	0.95	0.000
change following acquisitions (acquired facility) , exp($\beta + \tau + \gamma + \theta$)	0.91	0.87	0.94	0.000
difference in change following acquisitions†, exp($\gamma + \theta$)	0.99	0.94	1.05	0.857

Note: See **eTable7** for full regression model results. †The difference-in-difference estimate remained non-significant in an analysis of chain-owned facilities acquired as a part of DaVita's acquisition of Gambro and Fresenius's acquisition of Renal Care Group (DID estimate: 1.01; 95% CI 0.95 to 1.08).

eTable 6. Summary of Regression Results Examining Hospital Days per Patient-Year

**Un-Stratified Analysis (All Facility Ownership Types)**				
	Avg. Days	LCI	UCI	p-value
acquired vs. non-acquired facility	-1.23	-1.66	-0.80	0.000
change following acquisitions (non-acquired facility)	-2.05	-2.48	-1.61	0.000
change following acquisitions (acquired facility)	-0.89	-1.32	-0.46	0.000
difference in change following acquisitions	1.13	0.52	1.75	0.000
**Stratified Analysis**				
Independently-owned Facilities				
	Avg. Days	LCI	UCI	p-value
acquired vs. non-acquired facility	-2.92	-3.80	-2.03	0.000
change following acquisitions (non-acquired facility)	-2.63	-3.57	-1.68	0.000
change following acquisitions (acquired facility)	0.03	-0.82	0.89	0.939
difference in change following acquisitions	2.66	1.36	3.97	0.000
Chain-owned Facilities				
	Avg. Days	LCI	UCI	p-value
acquired vs. non-acquired facility	-0.72	-1.23	-0.22	0.005
change following acquisitions (non-acquired facility)	-1.92	-2.41	-1.43	0.000
change following acquisitions (acquired facility)	-1.20	-0.71	-1.69	0.000
difference in change following acquisitions†	0.69	-0.01	1.39	0.054

Note: Results are from estimated marginal effects. See **eTable8** for full regression model results. †The difference-in-difference estimate remained non-significant in an analysis of chain-owned facilities acquired as a part of DaVita's acquisition of Gambro and Fresenius's acquisition of Renal Care Group (DID estimate: 0.19 days; 95% CI -0.72 to 1.09)

© 2019 Erickson KF et al. *JAMA Network Open.*

eTable 7. Full Results from Cox Models Examining Association between Acquisitions and Mortality

	Un-Stratified Model			Stratified Model		
	HR	LCI	UCI	HR	LCI	UCI
facility acquired	0.97	0.94	1.00	0.90	0.84	0.95
period after acquisitions	0.89	0.86	0.92	0.80	0.74	0.86
chain-owned facility				0.96	0.91	1.01
Interaction terms						
acquisition*period after	1.02	0.98	1.07	1.15	1.04	1.26
chain-owned*acquired				1.11	1.04	1.20
chain-owned*period after				1.14	1.06	1.24
chain-owned*acquired*period after				0.87	0.78	0.96
demographic and socioeconomic						
Female	1.00	0.98	1.02	1.00	0.98	1.02
race (White is referent)						
Black	0.78	0.76	0.80	0.78	0.76	0.80
Native American	0.66	0.57	0.77	0.66	0.57	0.77
"Other"	0.65	0.61	0.70	0.65	0.61	0.70
Hispanic ethnicity	0.68	0.65	0.71	0.68	0.65	0.71
age (50-64 is referent)						
18-54 years	0.67	0.64	0.71	0.67	0.64	0.71
65-75 years	1.47	1.42	1.52	1.47	1.42	1.52
over 75 years	2.09	2.02	2.16	2.09	2.02	2.16
Uninsured at onset of dialysis	0.70	0.66	0.74	0.70	0.66	0.74
Medicaid eligible	0.98	0.96	1.01	0.98	0.96	1.01
Zip code median household income ($10K)	1.00	0.99	1.01	1.00	0.99	1.00
Zip code percent below poverty	1.00	1.00	1.00	1.00	1.00	1.00
Health						
Cancer	1.37	1.32	1.42	1.37	1.32	1.42
Heart failure	1.27	1.24	1.30	1.27	1.24	1.30
Cerebrovascular disease	1.12	1.08	1.16	1.12	1.08	1.16
Diabetes	0.99	0.96	1.01	0.99	0.96	1.01
Coronary disease	1.00	0.98	1.03	1.00	0.98	1.03
Drug or alcohol abuse	1.28	1.18	1.38	1.28	1.18	1.38
Immobility	1.77	1.71	1.83	1.77	1.71	1.83
eGFR -- ml/min/1.73m^2	1.03	1.03	1.04	1.03	1.03	1.04
Serum albumin level (>3.5 g/dL referent)						
< 2	2.13	2.02	2.25	2.13	2.02	2.25
2-3	1.59	1.55	1.64	1.59	1.55	1.64
3-3.5	1.30	1.26	1.35	1.30	1.26	1.35
Hemoglobin (>11 g/dL referent)						
< 8	1.04	0.99	1.09	1.04	0.99	1.09
8-9.5	1.05	1.01	1.09	1.05	1.01	1.09
9.5-11	1.05	1.02	1.08	1.05	1.02	1.08
Body Mass Index (>30 kg/m2 referent)						
< 18.5	1.80	1.72	1.90	1.80	1.72	1.90
18.5-25	1.34	1.30	1.38	1.34	1.30	1.38
25-30	1.11	1.07	1.14	1.11	1.07	1.14
Distance to dialysis facility - log miles	0.96	0.95	0.97	0.96	0.95	0.97
Market competition index (HHI) - 0.25 unit change	0.98	0.97	1.00	0.98	0.96	1.00
Population Density (metropolitan is referent)						
Micropolitan	0.97	0.92	1.02	0.97	0.92	1.02
Rural & small-town	0.99	0.94	1.05	1.00	0.95	1.05
Cohort Year (2004 is referent)						
05	1.00	0.95	1.04	0.99	0.94	1.04
06	0.99	0.94	1.03	0.97	0.92	1.01
07	0.93	0.89	0.98	0.92	0.87	0.96
08	0.94	0.88	1.01	0.95	0.88	1.02
09	0.95	0.89	1.01	0.94	0.88	1.00
10	0.90	0.84	0.96	0.89	0.83	0.95
11	0.85	0.79	0.90	0.84	0.79	0.90
12	0.80	0.76	0.85	0.79	0.75	0.84
13	0.80	0.75	0.86	0.79	0.74	0.85

© 2019 Erickson KF et al. *JAMA Network Open.*

Season (spring is referent)						
Summer	1.01	0.98	1.05	1.01	0.98	1.04
Fall	1.02	0.98	1.05	1.02	0.98	1.05
Winter	0.97	0.94	1.00	0.97	0.94	1.00

Note: 95% confidence intervals and p-values do not account for multiple comparisons.

eTable 8. Negative Binomial Models Examining Association between Acquisitions and Hospital Days

	Un-stratified model			Stratified Model		
	IRR	LCI	UCI	IRR	LCI	UCI
facility acquired	0.93	0.91	0.96	0.85	0.80	0.89
period after acquisitions	0.89	0.87	0.91	0.86	0.82	0.91
chain-owned facility				0.97	0.93	1.01
interaction terms						
acquisition*period after	1.07	1.03	1.10	1.16	1.08	1.25
chain-owned*acquired				1.14	1.07	1.20
chain-owned*period after				1.04	0.98	1.11
chain-owned*acquired*period after				0.89	0.82	0.97
demographic and socioeconomic						
Female	1.19	1.17	1.21	1.19	1.17	1.21
race (White is referent)						
Black	0.97	0.95	0.99	0.97	0.95	0.99
Native American	0.74	0.66	0.81	0.74	0.66	0.81
"Other"	0.79	0.75	0.83	0.79	0.75	0.83
Hispanic ethnicity	0.86	0.83	0.88	0.85	0.83	0.88
age (50-64 is referent)						
18-54 years	0.99	0.96	1.02	0.99	0.97	1.02
65-75 years	1.08	1.05	1.11	1.08	1.05	1.11
over 75 years	1.12	1.09	1.15	1.12	1.09	1.15
Uninsured at onset of dialysis	0.82	0.79	0.85	0.82	0.79	0.85
Medicaid eligible	1.16	1.13	1.18	1.15	1.13	1.18
Zip code median household income ($10K)	1.00	1.00	1.01	1.00	1.00	1.01
Zip code percent below poverty	1.00	1.00	1.00	1.00	1.00	1.00
Health						
Cancer	1.11	1.07	1.15	1.11	1.07	1.15
Heart failure	1.14	1.12	1.16	1.14	1.12	1.16
Cerebrovascular disease	1.13	1.09	1.16	1.13	1.10	1.16
Diabetes	1.15	1.13	1.17	1.15	1.13	1.17
Coronary disease	1.05	1.03	1.08	1.05	1.03	1.08
Drug or alcohol abuse	1.18	1.12	1.25	1.18	1.12	1.25
Immobility	1.55	1.50	1.60	1.55	1.51	1.60
eGFR -- ml/min/1.73m^2	1.03	1.03	1.03	1.03	1.03	1.03
Serum albumin level (>3.5 g/dL referent)						
< 2	1.97	1.87	2.07	1.97	1.87	2.07
2-3	1.49	1.45	1.53	1.49	1.45	1.53
3-3.5	1.24	1.21	1.27	1.24	1.21	1.27
Hemoglobin (>11 g/dL referent)						
< 8	1.08	1.04	1.11	1.08	1.04	1.11
8-9.5	1.10	1.07	1.13	1.09	1.06	1.13
9.5-11	1.08	1.05	1.10	1.08	1.05	1.10
Body Mass Index (>30 kg/m2 referent)						
< 18.5	1.28	1.23	1.34	1.28	1.23	1.34
18.5-25	1.12	1.09	1.14	1.12	1.09	1.14
25-30	1.02	1.00	1.05	1.02	1.00	1.05
Distance to dialysis facility - log miles	1.00	0.99	1.01	1.00	0.99	1.01
Market competition index (HHI) - 0.25 unit change	0.89	0.88	0.91	0.89	0.88	0.90
Population Density (metropolitan is referent)						
Micropolitan	0.93	0.90	0.97	0.93	0.90	0.97
Rural & small-town	0.86	0.83	0.90	0.87	0.83	0.90
Cohort Year (2004 is referent)						
05	1.01	0.97	1.05	1.01	0.97	1.05
06	0.99	0.96	1.03	0.98	0.94	1.01
07	0.98	0.94	1.02	0.97	0.93	1.01
08	1.00	0.94	1.06	1.00	0.95	1.06
09	0.92	0.87	0.96	0.91	0.87	0.96

10	0.87	0.83	0.91	0.86	0.82	0.91
11	0.87	0.83	0.92	0.87	0.83	0.91
12	0.79	0.76	0.83	0.79	0.76	0.82
13	0.77	0.73	0.81	0.77	0.73	0.81
Season (spring is referent)						
Summer	1.03	1.00	1.06	1.03	1.00	1.05
Fall	1.01	0.99	1.04	1.01	0.99	1.04
Winter	1.00	0.98	1.03	1.00	0.98	1.03
	IRR	LCI	UCI	IRR	LCI	UCI
Months since day 90 (1 is referent)						
2	0.95	0.94	0.97	0.95	0.94	0.97
3	0.91	0.90	0.93	0.91	0.90	0.93
4	0.88	0.86	0.89	0.88	0.86	0.89
5	0.85	0.83	0.87	0.85	0.83	0.87
6	0.85	0.83	0.86	0.85	0.83	0.86
7	0.83	0.81	0.85	0.83	0.81	0.85
8	0.81	0.79	0.83	0.81	0.79	0.83
9	0.80	0.78	0.82	0.80	0.78	0.82
10	0.78	0.76	0.81	0.78	0.76	0.81
11	0.77	0.75	0.79	0.77	0.75	0.79
12	0.77	0.75	0.79	0.77	0.75	0.79

IRR is incident rate ratio. 95% confidence intervals and p-values do not account for multiple comparisons.

© 2019 Erickson KF et al. *JAMA Network Open.*

eTable 9. Estimates from Models Using Alternative Controls.

Mortality:

coefficients pertaining to independently-owned facilities				
	HR Ratio	LCI	UCI	p-value
At a facility that will be (or has been) acquired	0.99	0.95	1.04	0.738
Change following acquisitions at non-acquired facilities	0.87	0.83	0.90	0.000
Change following acquisitions at acquired facilities	0.92	0.87	0.98	0.012
Difference in change among acquired facilities	1.06	0.99	1.15	0.093
**coefficients pertaining to differences in chain-owned facilities**				
	HR Ratio	LCI	UCI	p-value
At chain-owned facility	1.04	1.02	1.07	0.001
At a chain-owned facility that will be (or has been) acquired	1.04	1.00	1.09	0.049
Difference in post-acquisition change due to chain ownership	1.02	0.97	1.08	0.441
Modification of diff-in-diff estimate due to chain ownership	0.95	0.87	1.03	0.229

Note: Regression models adjust for all characteristics listed in **eTable7.**

Hospital days per patient-year:

coefficients pertaining to independently-owned facilities				
	Hosp days diff.	LCI	UCI	p-value
At a facility that will be (or has been) acquired	-1.41	-2.21	-0.60	0.001
Change following acquisitions at non-acquired facilities	-2.00	-2.63	-1.36	0.000
Change following acquisitions at acquired facilities	0.55	-0.68	1.77	0.382
Difference in change among acquired facilities	2.07	0.83	3.32	0.001
**coefficients pertaining to differences in chain-owned facilities**				
	Hosp days diff.	LCI	UCI	p-value
At chain-owned facility	0.56	0.10	1.02	0.018
At a chain-owned facility that will be (or has been) acquired	0.91	-0.03	1.85	0.059
Difference in post-acquisition change due to chain ownership	-0.31	-0.99	0.38	0.379
Modification of diff-in-diff estimate due to chain ownership	-0.94	-2.36	0.49	0.198

Note: Regression models adjust for all characteristics listed in **eTable8**

Summary of Findings from Alternative Controls:

When comparing the hazards of death using the alternative control, acquisitions were no longer independently associated with mortality in the pre-acquisition (i.e. baseline) period at independently-owned facilities (HR 0.99; 95% CI 0.95 to 1.04). Declines over time in mortality at independently-owned acquired facilities continued to be slower than declines in mortality at independently-owned facilities that were not acquired. Acquisitions were associated with a (relatively) higher rate of death (diff-in-diff estimate 1.06; 95% CI 0.99 to 1.15). This 6% difference is less than the 15% difference observed in our primary analysis, and was of marginal statistical significance (p=0.09). Specifically, the magnitude of the difference-in-differences estimate for independently-owned facilities was 60% smaller in the alternative control compared to the primary model of survival.

A similar pattern emerged when comparing changes over time in hospitalization days at independently-owned facilities using the alternative control group. Among independently-owned facilities, acquisitions continued to be independently associated with a slower decline in hospital days (diff-in-diff estimate 2.1; 95% CI 0.8 to 3.3. This was only slightly smaller in magnitude than the difference of 2.7 (95% CI 1.4 to 4.0) observed in the primary analysis. Specifically, the magnitude of the difference-in-differences estimate for independently-owned facilities was 22% smaller in the alternative control compared to the primary model of hospitalizations.

© 2019 Erickson KF et al. *JAMA Network Open.*

eTable10. Examining for "Regression to the Mean"

Mortality

	independently-owned								
	2-yrs prior to baseline			baseline period			post-acquisition		
	HR	LCI	UCI	HR	LCI	UCI	HR	LCI	UCI
acquired	0.92	0.86	0.98	0.9	0.85	0.96	0.83	0.77	0.89
non-acquired	1.02	0.97	1.09	index			0.8	0.74	0.86
	**chain-owned**								
	2-yrs prior to baseline			baseline period			post-acquisition		
	HR	LCI	UCI	HR	LCI	UCI	HR	LCI	UCI
acquired	0.89	0.8	0.99	0.96	0.91	1.02	0.87	0.83	0.92
non-acquired	1.02	0.97	1.07	0.96	0.91	1.01	0.88	0.83	0.93

Note: Regression model controls for all covariates listed in **eTable7**.

Hospital days per patient-year

	independently-owned								
	2-yrs prior to baseline			baseline period			post-acquisition		
	Hosp Days	LCI	UCI	Hosp Days	LCI	UCI	Hosp Days	LCI	UCI
Acquired	15.3	14.7	15.9	17.0	16.4	17.5	17.0	16.4	17.6
non-acquired	17.7	17.1	18.3	19.5	18.8	20.2	16.4	15.7	17.1
	**chain-owned**								
	2-yrs prior to baseline			baseline period			post-acquisition		
	Hosp Days	LCI	UCI	Hosp Days	LCI	UCI	Hosp Days	LCI	UCI
Acquired	17.2	16.9	17.6	17.6	17.3	18.0	16.4	16.0	16.7
non-acquired	18.3	18.0	18.6	18.7	18.3	19.0	16.3	16.0	16.7

Note: See **eMethods, "Examining Regression to the Mean"** for detailed methods. Displayed results are estimated marginal effects following a negative binomial regression model. Models controls for all covariates listed in **eTable8**.

Summary of Findings from Examination of "Regression to the Mean"

In an examination of mortality, there was a 10% lower hazard of death in independently-owned acquired facilities compared to independently-owned non-acquired facilities in the baseline period prior to acquisitions. This is similar in magnitude to the 10.4% "gap" in mortality observed at independently-owned facilities in our primary analysis. Similar to findings from our primary analysis, this mortality difference narrowed following acquisitions, as acquired facilities experienced smaller declines over time in adjusted mortality (difference-in-difference estimate for independently-owned facilities: 1.15; 95% CI 1.05 to 1.27). In contrast to the post-acquisition period, baseline differences in mortality were similar when looking back to the 2-years prior to entry into each cohort. Compared to patients at non-acquired facilities who initiated dialysis in the baseline period (who served as the "index"), the estimated hazard of death at acquired facilities was 10% lower in the baseline period and 8% lower in the 2-years prior to baseline. The p-value testing whether differences in adjusted mortality between acquired and non-acquired facilities changed in magnitude in the 2-years prior to acquisitions was 0.92.

When examining hospitalization days in the baseline period, patients at independently-owned facilities that were acquired had 2.5 (95% CI -3.3 to -1.6) fewer hospitalization days per patient-year compared to those at non-acquired facilities. Similar to findings from our primary analysis, this difference narrowed to 0.6 in the period following acquisitions (p-value for difference-in-difference estimate of <0.001). Yet, the difference in hospitalization days between acquired and non-acquired independently-owned facilities remained virtually unchanged (at 2.4 fewer hospital days) in the 2-years prior to acquisitions. The p-value testing the significance of this difference was 0.84.

© 2019 Erickson KF et al. *JAMA Network Open.*

Together, these findings suggest that slower declines in health outcomes following acquisitions at acquired *versus* non-acquired facilities are unlikely to be explained by random chance. Differences in health outcomes between acquired and non-acquired facilities persisted when looking back in time, and only narrowed following acquisitions.

eTable11. Assessing Differences in Comorbidities among Comparison Groups

Outcomes predicted from observed changes in case-mix and geography

**1-year mortality**				**Hospitalization days per patient-year:**		
Independently-owned				Independently-owned		
	pre-acquisition	post-acquisition	diff	pre-acquisition	post-acquisition	diff
Acquired	27.6	27.6	0.0	20.0	19.6	-0.4
Non-acquired	24.5	24.4	-0.1	21.2	21.1	-0.1
Chain-owned				Chain-owned		
	pre-acquisition	post-acquisition	diff	pre-acquisition	post-acquisition	diff
Acquired	25.2	25.4	0.2	18.8	18.8	0.0
Non-acquired	23.2	22.9	-0.2	19.2	18.8	-0.4

Variation in in 1-year mortality and hospital days predicted from observed differences among patient groups in geographic and patient characteristics was relatively small, ranging from 0 to 0.4 in hospital days per patient-year and 0.0 to 0.2 in the 1-year probability of death. While these findings do not directly address unobserved differences in case mix, they suggest that differences in patient and geographic characteristics did not lead to substantial changes in health outcomes.

© 2019 Erickson KF et al. *JAMA Network Open.*

eFigure 1. Study Selection Diagram

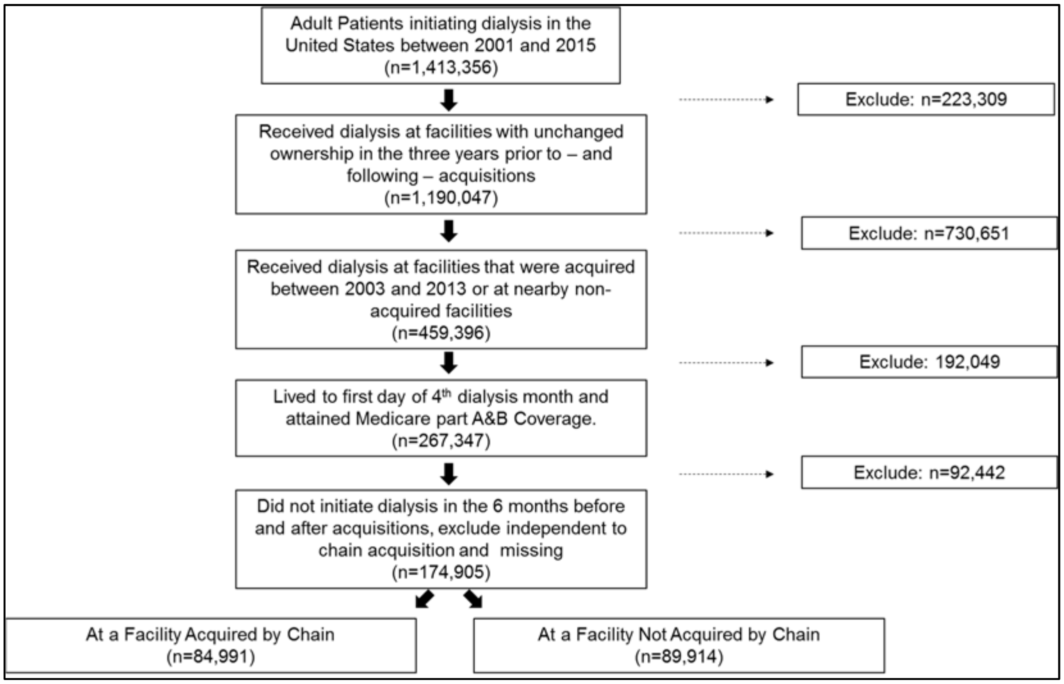

eFigure2. Unadjusted One-Year Probabilities of Death

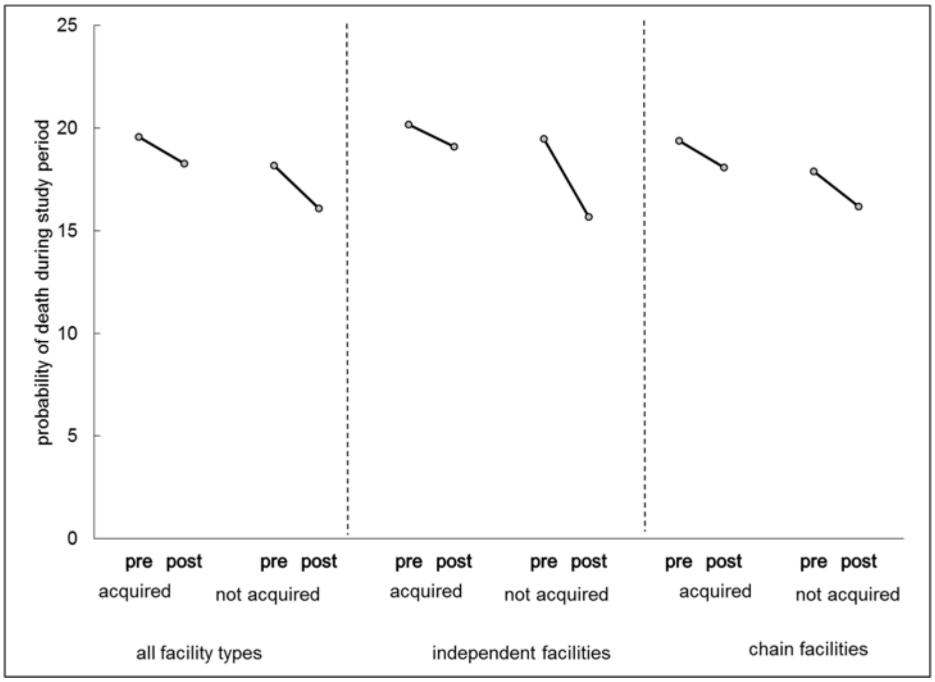

Note: Death during study period does not include death following censoring. P-values for differences in probabilities of death in pre-acquisition periods among acquired vs. non acquired facilities were <0.001 at All Facilities and at Chain-owned facilities. Probability of death at independently-owned facilities prior to acquisitions was 20.2% at acquired facilities and 19.5% at non-acquired facilities (p-value for difference-0.21)

© 2019 Erickson KF et al. *JAMA Network Open.*

eFigure3. Percentage Point improvements in Unadjusted Mortality Rates in the Period after Acquisitions

Figure 3a. All Facilities

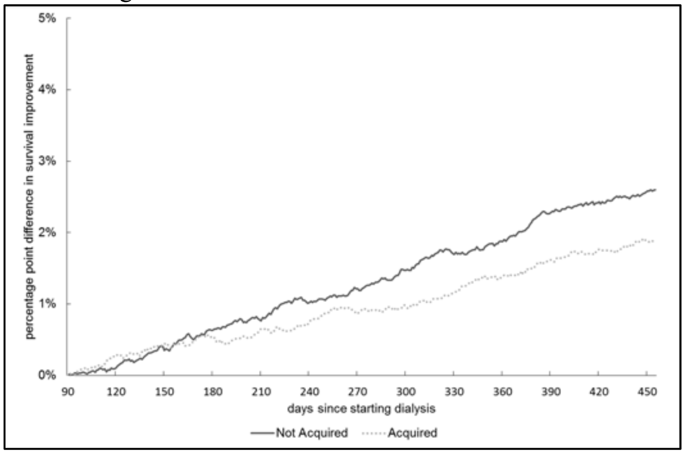

Figure 3b. Independent Facilities Prior to Acquisitions

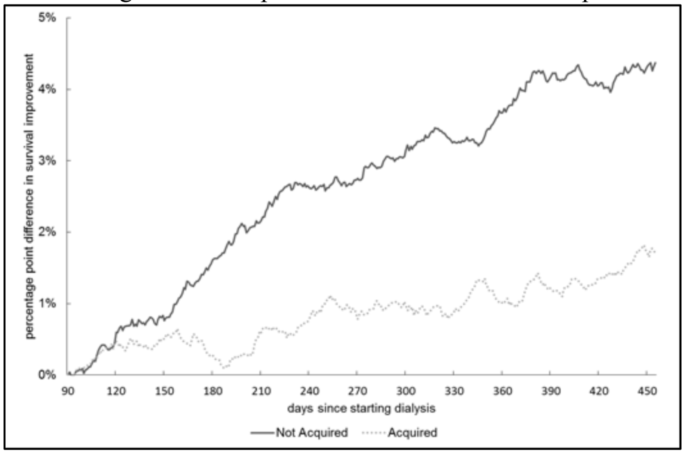

Figure 3c. Chain Facilities Prior to Acquisitions

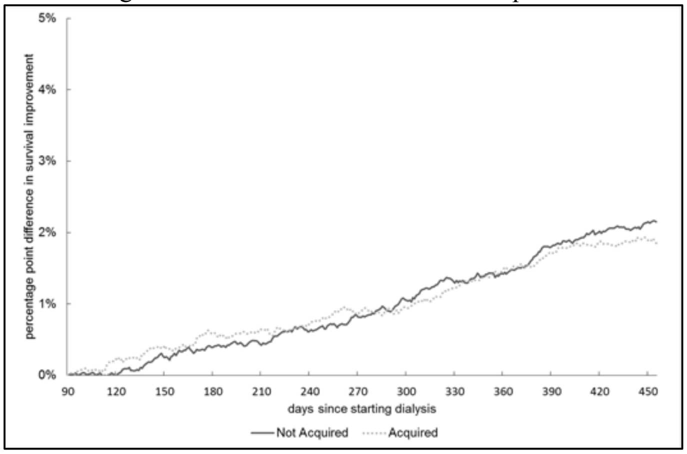

Note: Unadjusted improvements in rate of death were obtained from the difference of the cumulative probability of survival (using Kaplan Meier method) at each day among patients starting dialysis before versus after acquisitions.

© 2019 Erickson KF et al. *JAMA Network Open.*

eFigure4. Unadjusted Hospital Days per Patient-Year

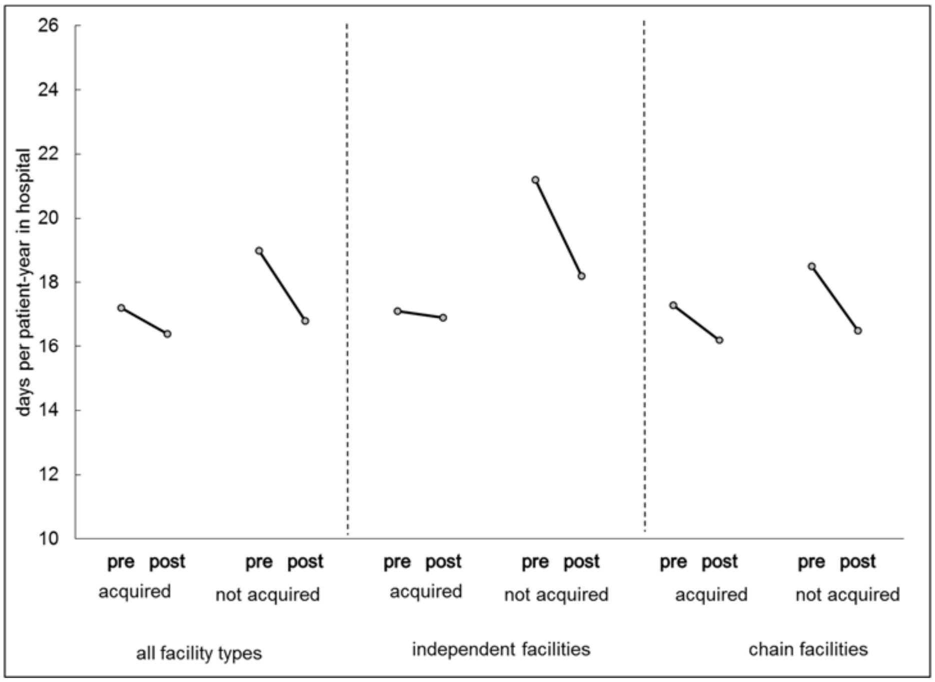

Note: Unadjusted hospital days at independently-owned facilities that were acquired declined by 0.2 days following acquisitions. This change was not statistically significant (p=0.3). Among chain-owned facilities prior to acquisitions, hospitalization days per patient-year were 17.3 at acquired and 18.5 at non-acquired facilities. Following acquisitions, hospital days declined by 1.1 (p<0.001) at acquired facilities *versus* 2.0 (p<0.001) at non-acquired facilities.

© 2019 Erickson KF et al. *JAMA Network Open.*

eFigure5. Sensitivity Analyses Focusing on Mortality

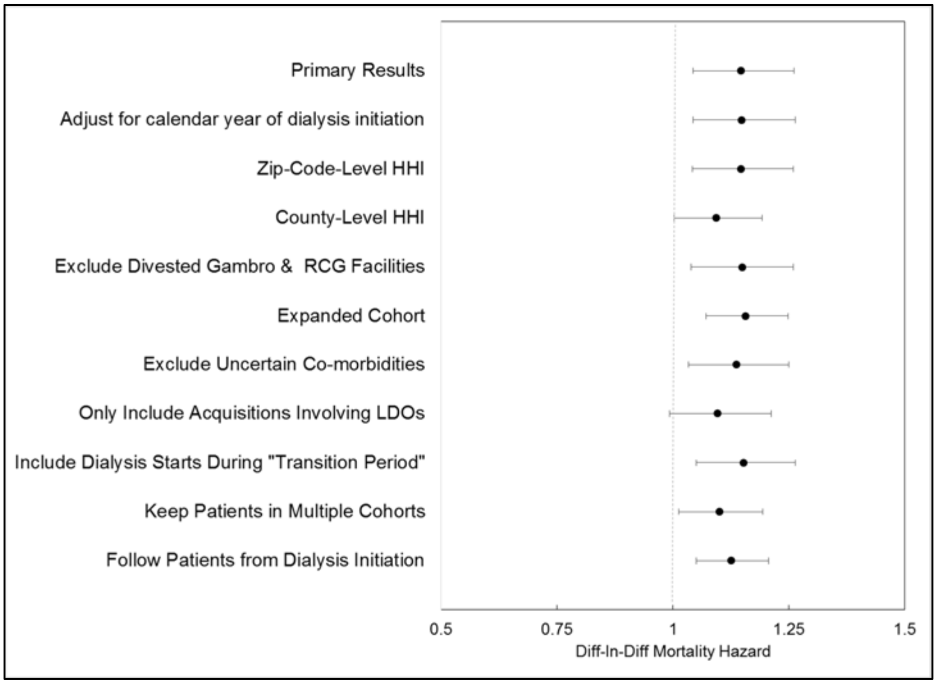

Detailed Results of Sensitivity Analyses Involving Mortality:

	Adjust for calendar year of dialysis initiation				Exclude Uncertain Co-morbidities			
	HR	LCI	UCI	p-value	HR	LCI	UCI	p-value
Acquired vs. non-acquired facility	0.90	0.84	0.95	0.001	0.90	0.85	0.96	0.001
Change following acquisition (independent facilities)	0.85	0.77	0.94	0.002	0.80	0.74	0.86	0.000
Diff-in-diff (independently-owned facilities)	1.15	1.04	1.26	0.004	1.14	1.03	1.25	0.008
Modification of diff-in-diff at chain-owned facilities	0.87	0.78	0.98	0.016	0.88	0.79	0.98	0.020
	Zip-Code-Level HHI				Only Include Acquisitions Involving LDOs			
	HR	LCI	UCI	p-value	HR	LCI	UCI	p-value
Acquired vs. non-acquired facility	0.90	0.84	0.95	0.001	0.93	0.86	1.00	0.052
Change following acquisition (independent facilities)	0.80	0.74	0.86	0.000	0.87	0.80	0.95	0.001
Diff-in-diff (independently-owned facilities)	1.15	1.04	1.26	0.005	1.10	0.99	1.21	0.070
Modification of diff-in-diff at chain-owned facilities	0.87	0.78	0.97	0.009	0.90	0.80	1.01	0.073
	County-Level HHI				Includes Dialysis During "Transition Period"			
	HR	LCI	UCI	p-value	HR	LCI	UCI	p-value
Acquired vs. non-acquired facility	0.91	0.86	0.97	0.002	0.89	0.84	0.95	0.000
Change following acquisition (independent facilities)	0.83	0.78	0.88	0.000	0.80	0.75	0.86	0.000
Diff-in-diff (independently-owned facilities)	1.09	1.00	1.19	0.043	1.15	1.05	1.26	0.003
Modification of diff-in-diff at chain-owned facilities	0.91	0.83	1.01	0.072	0.86	0.77	0.96	0.005
	Exclude Divested Gambro & RCG Facilities				Keep Patients in Multiple Cohorts			
	HR	LCI	UCI	p-value	HR	LCI	UCI	p-value
Acquired vs. non-acquired facility	0.90	0.84	0.95	0.001	0.90	0.85	0.95	0.000

© 2019 Erickson KF et al. *JAMA Network Open.*

Change following acquisition (independent facilities)	0.80	0.74	0.86	0.000	0.82	0.78	0.86	0.000
Diff-in-diff (independently-owned facilities)	1.15	1.04	1.26	0.005	1.10	1.01	1.19	0.023
Modification of diff-in-diff at chain-owned facilities	0.96	0.91	1.01	0.139	0.90	0.82	0.99	0.032
	Expanded Mortality Cohort				Follow Patients from Dialysis Initiation			
	HR	LCI	UCI	p-value	HR	LCI	UCI	p-value
Acquired vs. non-acquired facility	0.90	0.85	0.94	0.000	0.89	0.85	0.93	0.000
Change following acquisition (independent facilities)	0.80	0.76	0.85	0.000	0.84	0.80	0.88	0.000
Diff-in-diff (independently-owned facilities)	1.16	1.07	1.25	0.000	1.13	1.05	1.21	0.001
Modification of diff-in-diff at chain-owned facilities	0.87	0.80	0.95	0.001	0.89	0.82	0.96	0.004

Note: All models control for covariates listed in **eTable7**. "Modification of diff-in-diff at chain-owned facilities" is the estimated coefficient of 'θ' in the stratified regression model.

eFigure6. Sensitivity Analyses Focusing on Hospital Days per Patient-Year

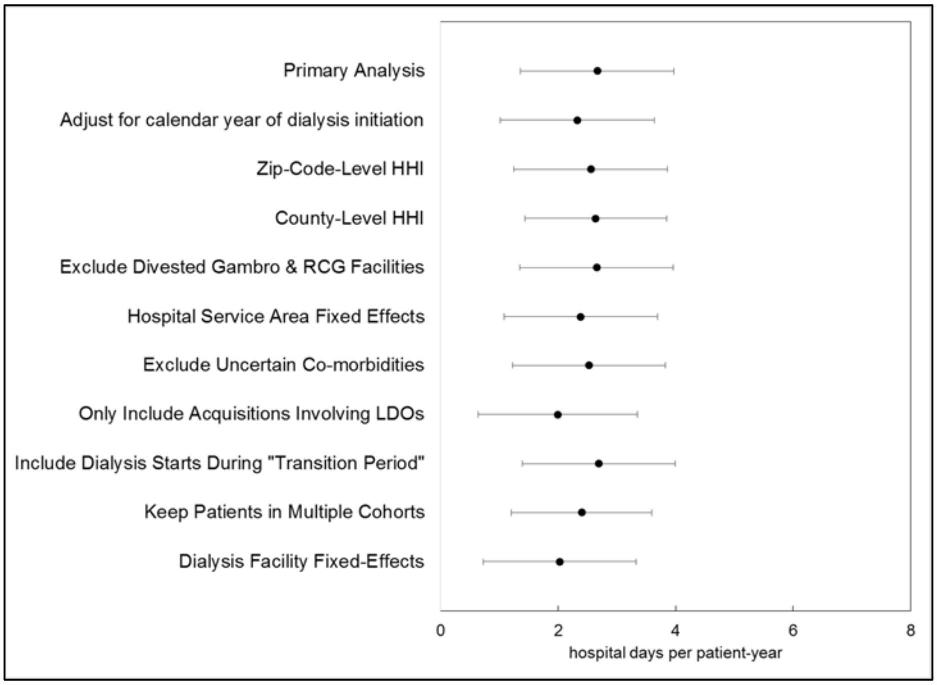

Detailed Results of Sensitivity Analysis Involving Hospital Days:

	Adjust for calendar year of dialysis start				Exclude Uncertain Co-morbidities			
	Hospital Days	LCI	UCI	p-value	Hospital Days	LCI	UCI	p-value
Acquired vs. non-acquired facility	-2.7	-3.6	-1.8	0.000	-2.9	-3.8	-2.0	0.000
Change following acquisition (independent facilities)	2.0	0.7	3.4	0.003	-2.6	-3.5	-1.6	0.000
Diff-in-diff (independently-owned facilities)	2.3	1.0	3.6	0.001	2.5	1.2	3.8	0.000
Modification of diff-in-diff at chain-owned facilities	-1.8	-3.3	-0.3	0.019	-1.8	-3.3	-0.3	0.017
	Zip-Code-Level HHI				Only Include Acquisitions Involving LDOs			
	Hospital Days	LCI	UCI	p-value	Hospital Days	LCI	UCI	p-value
Acquired vs. non-acquired facility	-2.8	-3.7	-1.9	0.000	-2.6	-3.7	-1.5	0.000
Change following acquisition (independent facilities)	-2.6	-3.6	-1.7	0.000	-2.2	-3.3	-1.1	0.000
Diff-in-diff (independently-owned facilities)	2.6	1.2	3.9	0.000	2.0	0.6	3.3	0.004

© 2019 Erickson KF et al. *JAMA Network Open.*

Modification of diff-in-diff at chain-owned facilities	-1.9	-3.3	-0.4	0.013	-1.9	-3.4	-0.4	0.016
	County-Level HHI				Include Dialysis Starts During Transition Period			
	Hospital Days	LCI	UCI	p-value	Hospital Days	LCI	UCI	p-value
Acquired vs. non-acquired facility	-2.9	-3.7	-2.1	0.000	-3.0	-3.8	-2.1	0.000
Change following acquisition (independent facilities)	-2.5	-3.3	-1.7	0.000	-2.7	-3.6	-1.7	0.000
Diff-in-diff (independently-owned facilities)	2.6	1.4	3.8	0.000	2.7	1.4	4.0	0.000
Modification of diff-in-diff at chain-owned facilities	-2.0	-3.4	-0.6	0.004	-2.0	-3.5	-0.5	0.008
	Exclude Divested Gambro & RCG Facilities				Keep Patients in Multiple Cohorts			
	Hospital Days	LCI	UCI	p-value	Hospital Days	LCI	UCI	p-value
Acquired vs. non-acquired facility	-2.9	-3.8	-2.0	0.000	-3.2	-4.1	-2.4	0.000
Change following acquisition (independent facilities)	-2.6	-3.6	-1.7	0.000	-2.5	-3.2	-1.8	0.000
Diff-in-diff (independently-owned facilities)	2.7	1.3	4.0	0.000	2.4	1.2	3.6	0.000
Modification of diff-in-diff at chain-owned facilities	-0.6	-1.3	-0.1	0.098	-1.8	-3.1	-0.4	0.010
	Hospital Service Area Fixed Effects				Dialysis Facility Fixed-Effects			
	Hospital Days	LCI	UCI	p-value	Hospital Days	LCI	UCI	p-value
Acquired vs. non-acquired facility	-1.8	-2.9	--0.8	0.001	1.3	-1.0	3.6	0.272
Change following acquisition (independent facilities)	-3.1	-4.1	-2.1	0.000	-2.7	-3.7	-1.7	0.000
Diff-in-diff (independently-owned facilities)	2.4	1.1	3.7	0.000	2.0	0.7	3.3	0.002
Modification of diff-in-diff at chain-owned facilities	-1.9	-3.3	-0.4	0.012	-1.5	-2.9	0.0	0.047

Note: All models control for covariates listed in **eTable8**. “Modification of diff-in-diff at chain-owned facilities” is the estimated coefficient of ‘θ’ in the stratified regression model.

Summary of Sensitivity Analysis Results:

Our primary study findings were not sensitive to the alternative model specifications listed above. In particular, the difference-in-differences estimates involving patients at independently-owned facilities remained close to the values observed in our primary cohorts. Acquisitions did not consistently predict changes in health outcomes at chain-owned facilities in our primary analyses, and the predicted effects of acquisitions on chain-owned facilities varied somewhat across different model specifications.

In an additional sensitivity analysis **(Results not shown)**, acquisitions of Gambro by DaVita and Renal Care Group by Fresenius were not associated with mortality or hospitalizations. This is consistent with the broader finding that acquisitions of chain-owned facilities were not associated with health outcomes.

We did not find evidence that the estimated effect of acquisitions changed following enactment of ESRD payment reform and we did not observe heterogeneity over time in the estimated effect of acquisitions on hospital days and mortality. **(Results not shown)**

© 2019 Erickson KF et al. *JAMA Network Open.*
